# Vascular Endothelial Growth Factor (VEGF) Bioavailability Regulates Angiogenesis and Intestinal Stem and Progenitor Cell Proliferation during Postnatal Small Intestinal Development

**DOI:** 10.1371/journal.pone.0151396

**Published:** 2016-03-15

**Authors:** Christopher R. Schlieve, Salvador Garcia Mojica, Kathleen A. Holoyda, Xiaogang Hou, Kathryn L. Fowler, Tracy C. Grikscheit

**Affiliations:** 1 Developmental Biology and Regenerative Medicine Program, The Saban Research Institute at Children’s Hospital Los Angeles, Los Angeles, California, United States of America; 2 Department of Surgery, Division of Pediatric Surgery, Children’s Hospital Los Angeles, Los Angeles, California, United States of America; 3 Department of Biology, California State University San Bernardino, San Bernardino, California, United States of America; Medical College of Wisconsin, UNITED STATES

## Abstract

**Background:**

Vascular endothelial growth factor (VEGF) is a highly conserved, master regulatory molecule required for endothelial cell proliferation, organization, migration and branching morphogenesis. *Podocoryne carnea* and *drosophila*, which lack endothelial cells and a vascular system, express VEGF homologs, indicating potential roles beyond angiogenesis and vasculogenesis. The role of VEGF in the development and homeostasis of the postnatal small intestine is unknown. We hypothesized regulating VEGF bioavailability in the postnatal small intestine would exhibit effects beyond the vasculature and influence epithelial cell stem/progenitor populations.

**Methods:**

VEGF mutant mice were created that overexpressed VEGF in the brush border of epithelium via the villin promotor following doxycycline treatment. To decrease VEGF bioavailability, sFlt-1 mutant mice were generated that overexpressed the soluble VEGF receptor sFlt-1 upon doxycycline administration in the intestinal epithelium. Mice were analyzed after 21 days of doxycycline administration.

**Results:**

Increased VEGF expression was confirmed by RT-qPCR and ELISA in the intestine of the VEGF mutants compared to littermates. The VEGF mutant duodenum demonstrated increased angiogenesis and vascular leak as compared to littermate controls. The VEGF mutant duodenum revealed taller villi and increased Ki-67-positive cells in the transit-amplifying zone with reduced Lgr5 expression. The duodenum of sFlt-1 mutants revealed shorter villi and longer crypts with reduced proliferation in the transit-amplifying zone, reduced expression of Dll1, Bmp4 and VE-cadherin, and increased expression of Sox9 and EphB2.

**Conclusions:**

Manipulating VEGF bioavailability leads to profound effects on not only the intestinal vasculature, but epithelial stem and progenitor cells in the intestinal crypt. Elucidation of the crosstalk between VEGF signaling in the vasculature, mesenchyme and epithelial stem/progenitor cell populations may direct future cell therapies for intestinal dysfunction or disease.

## Introduction

Considered the master regulatory cytokine of vasculogenesis and angiogenesis, vascular endothelial growth factor (VEGF) is evolutionarily highly conserved and identified throughout all developmental stages. The mammalian VEGF family of ligands, VEGF-A, VEGF-B, VEGF-C, VEGF-D and placental growth factor (PlGF), bind to homodimers or heterodimers of the transmembrane tyrosine kinase receptors VEGFR1 (Flt-1), VEGFR2 (Flk/KDR), or VEGFR3 (Flt-4). In mice, VEGF expression peaks embryonically in the yolk sac and embryo and steadily declines in all organs in adults [1]. The loss of a single allele leads to *in utero* lethality between embryonic days 11 and 12 [2, 3]. In contrast, VEGF expression in sheep jejunum is elevated in term animals compared to fetal stages, suggesting a greater role during postnatal development [4]. Complex regulation of vasculogenesis and angiogenesis occurs through alternative splicing of VEGF ligands and receptors, producing pro-angiogenic and anti-angiogenic isoforms that are implicated in a host of healthy and diseased states [5]. In mice, alternative splicing of VEGFR1 truncates the intracellular domain and creates a soluble receptor sFlt-1, which has a high affinity for VEGF-A, thereby reducing its bioavailability [6].

VEGF signaling biodiversity leads to complex regulation of not only vasculogenesis and angiogenesis, but cell proliferation, migration, survival and permeability [5]. VEGF regulates branching morphogenesis in mammalian vasculature, neurons, lung and pancreas epithelium [7, 8]. In human and mouse, VEGF-C activates quiescent neural stem cells through VEGFR3 to enter the cell cycle and generate progenitor cells [9]. Additionally, VEGF-A influences differentiation of mesenchymal stem cells into osteoblasts and adipocytes by regulating the levels of the osteoblast and adipocyte transcription factors Runx2 and PPARγ, respectively [10]. These observations suggest that VEGF has a crucial role in regulation of stem and progenitor cell populations, independent of vasculogenesis.

The presence of VEGF in the gastrointestinal system of organisms lacking vascular systems suggests that VEGF may play a crucial role in the maintenance of homeostasis in multiple organ systems, including the gastrointestinal tract. Despite a lack of endothelium and blood cells, jellyfish (*podocoryne carnea*) express a VEGF homolog in their gastrovascular system, suggesting a role for VEGF in the development of the gastrointestinal system [11]. Intrinsic platelet-derived growth factor, also known as vascular endothelial growth factor-like factor (Pvf), is required for homeostasis and differentiation of intestinal stem cells in the posterior midgut of *Drosophila*. Hyperactivity of Pvf/Pvr drives intestinal dysplasia, supporting its role as a regulator of intestinal stem cells [12, 13]. The necessity of VEGF homologs in gastrointestinal development and their augmentation causing dysplasia suggest an interaction between VEGF and the intestinal stem cell niche.

Although embryonic VEGF augmentation in gastrointestinal epithelium has been implicated in the development of neoplasia [14], the role of VEGF in postnatal small intestinal development and homeostasis is currently unknown. The goal of this study is to elucidate the role of VEGF on the postnatal intestinal stem cell niche in a murine model. Triple transgenic mice were generated with the ability to augment VEGF in epithelial cells of the gastrointestinal tract in an inducible manner via the villin promoter (VEGF-Tg) or decrease VEGF bioavailability through villin-driven overexpression of an inducible soluble VEGFR-1 (sFlt-Tg).

## Methods

### Generation of Villin^Cre^/rtTA^flox/flox^/tet(o)VEGF and Villin^Cre^/rtTA^flox/flox^/tet(o)s-Flt1 (sFlt-1 mutants) mutant mice, Transgene PCR, and Tissue Collection

Transgenic mice on a C57/B6 background were maintained according to the animal care facility protocols of the institution with approval by the Children's Hospital Los Angeles Institutional Animal Care and Use Committee. All mice were housed in a controlled environment in clean cages, fed mouse chow or doxycycline chow *ad libitum* with an unlimited source of fresh water. Tail clips were collected from mice that were P14 or older under isofluorane anesthesia and were euthanized under CO_2_ exposure at P21.

Triple transgenic Villin^Cre^/rtTA^flox/flox^/tet(o)VEGF mutant mice (VEGF mutants) or Villin^Cre^/rtTA^flox/flox^/tet(o)s-Flt1 mutant mice (sFlt-1 mutants) were established. Intestine-specific VEGF or sFlt-1 overexpression was inducible with the administration of oral doxycycline. Villin^Cre^ mice [15] were mated with tet(o) VEGF [16] or tet(o) sFlt-1 [17] mice. Those positive for both genes were crossed with homozygous rtTA^flox/flox^ mice [18]. After birth of a litter, the mother was fed 625 mg/kg doxycycline chow (Harlan; Cat# TD.110720) *ad libitum*. The doxycycline chow induced overexpression of VEGF or sFlt-1 in the pups via the mother’s breast milk.

The mice were genotyped by polymerase chain reaction (PCR) at P14. The pups were placed under general anesthesia and a small tail clip was acquired. The specimen was placed into Direct PCR Tail reagent (Viagen Biotech; Cat# 102-T) with 1:100 Proteinase K Solution (Invitrogen; Cat# 25530049) and moved to the 55°C incubator overnight. The temperature was increased to 85°C in the morning for one hour, then returned to room temperature. The genotyping PCR mix consisted of 10 μL MyTaq Red Mix (Bioline; Cat# BIO-25043), 0.1 μL 100 μM forward (F) primer, 0.1 μL 100 μM reverse (R) primer (Eurofins MWG Operon, Table 1), 8.8 μL RNase-free water, and 1 μL mouse DNA for a total of 20 μL PCR reaction. This was placed in a 0.2 μL PCR tube. PCR was performed in a thermocycler with the temperature recommended by the manufacturer for the MyTaq Red Mix and appropriate annealing temperatures (Table 1). A 1% agarose (Denville Scientific; Cat# CA3510-8) with 1:1000 ethidium bromide (Promega Corporation; Cat# H5041) electrophoresis gel was prepared and 10 μL of mix was run at 100V for 30 minutes and visualized under a UV light. The mice that contained one or two alleles were named littermates (LM) and those containing all three alleles were termed mutants.

**Table 1:** Genotyping Primer Sequences and Annealing Temperatures Employed.

Primers	Sequence	Annealing Temp (°C)
Villin-Cre F	5’- CAAGCCTGGCTCGACGGCC -3’	62
Villin-Cre R	5’- CGCGAACATCTTCAGGTTCT-3’	62
rtTA R	5’- AAGACCGCGAAGAGTTTGTC -3’	58
Rosa26 F	5- GAGTTCTCTGCTGCCTCCTG -3’	58
Rosa26 R	5’- CGAGGCGGATACAAGCAATA-3’	58
Tet(o)-VEGF F	5’- CGCGAAGCTTCCATGCTCTCTTGGGT -3’	55
Tet(o)-VEGF R	5’- CGCGGATATCACCTTGGCTTGTCACA -3’	55
Tet(o)-sFlt-1 F	5’- CGACTCACTATAGGGAGACCC -3’	55
Tet(o)-sFlt-1 R	5’- TGGCCTGCTTGCATGATGTGCTGG -3’	55

The above are nucleotide sequences for the listed RT-qPCR genes along with the appropriate annealing temperatures according to the manufacturer employed to confirm transgenic strains.

The duodenum of the mice was harvested at P21. After euthanization of the mice in a CO_2_ chamber, three matched 5 cm segments of proximal duodenum were harvested. Samples were placed in 10% formalin (Fisher Scientific; Cat#305–510), RNAlater (Sigma-Aldrich; R0901), or flash frozen on dry ice and placed in the -80°C freezer.

### Histology and Immunofluorescence

Formalin-fixed tissue was embedded in paraffin and sectioned at 5 μm. The tissue was dehydrated in 30% EtOH for 30 minutes, 50% EtOH for 45 minutes, 70% EtOH for 45 minutes, 95% EtOH for 1 hour, 100% EtOH for 1 hour twice. The tissue was then cleared with toluene twice for 45 minutes, then in 1:1 ratio of paraffin and toluene overnight at 65°C. The following morning the tissue was placed in pure paraffin for 1 hour twice and fixed in the cassette on a cooling plate to solidify. The tissue was then sectioned at 5 μm. Frozen sections were procured for confocal microscopy to evaluate angiogenesis after intracardiac FITC-dextran labeling of the vasculature. Tissue was protected from light and placed in 4% paraformaldehyde overnight at 4°C. The following day, tissue was placed in 30% sucrose until it sank to the bottom the tube. The tissue was then transferred to a 1:1 mixture of 30% sucrose:OTC compound overnight at 4°C. Tissue was subsequently transferred to OTC compound and slowly frozen at -20°C until the OTC was frozen. Blocks were stored at -80°C until sectioning. Frozen tissue was sectioned at 60 μm immunofluorescence staining with DAPI was performed as describe below.

Hematoxylin and eosin (H&E) staining was performed on the sectioned tissue. Slides were placed in Histochoice (Amresco; H103) twice for 2 minutes, then in 100% EtOH for 2 minutes. The slides were hydrated in 70% EtOH for 30 seconds, 50% EtOH for 30 seconds, 30% EtOH for 30 seconds, and H_2_O for 2 minutes. The slides were placed in hematoxylin for 15 seconds and H_2_O until clear, followed by eosin for 5 seconds for counterstain. The slides were dehydrated in 90% EtOH for 1 minute, 100% EtOH for 1 minute and Histochoice for 2 minutes. The slides were imaged at 20x magnification on a brightfield microscope. Single and multiple clusters of red blood cells (RBCs) were counted by blind observers as previously described [14]. The villus length, crypt depth, circumference and number of crypts per intestinal length were measured by a trained blinded observer with ImageJ [19].

Immunofluorescence was performed to locate terminal cell markers and proliferative markers. The slides were placed in Histochoice for 10 minutes twice. The slides were rehydrated by soaking them sequentially in 100% EtOH, 90% EtOH, 75% EtOH, 50% ETOH, and 30% EtOH for 5 minutes each. A low pH, citrate-based Antigen Unmasking Solution (Vector; H-3300) was used to retrieve antigens in the microwave. The microwave heated the solution for 4 minutes at 50% three times, with 30 seconds in between each heating session. The solution was cooled to room temperature for 30 minutes and washed in phosphate-buffered saline (Gibco; Cat#10010) with 0.1% Tween (Amresco; Cat# 9005-64-5) (PBS-T) for 5 minutes. Universal blocking solution (1% BSA, 0.1% cold fish skin gelatin, 0.5% Triton-X 100 and 1x PBS) with 2% goat serum (Sigma-Aldrich; Cat# G9023) was applied for 30 minutes at room temperature. Primary antibodies were diluted (Table 2) in universal blocking solution with 2% goat serum and placed on tissue overnight at 4°C. In the morning, tissues were washed in PBS-T for 5 minutes three times. Appropriate Cy3 or Cy5 secondary antibodies diluted in PBS with 0.05% Tween (Table 2) and applied to tissue for 1 hour at room temperature. The slides were again washed with PBS-T for 5 minutes three times. The sections were mounted on Vectashield with DAPI (Vector; Cat# H-1200) and visualized under the fluorescent microscope.

**Table 2:** Immunofluorescence Antibodies Applied for Staining.

Antibody	Antibody	Dilution	Company
Chromogranin A	Primary	1:100	Abcam
Lysozyme	Primary	1:100	DakoCytomation
Villin	Primary	1:100	Santa Cruz Biotechnology
Mucin 2	Primary	1:50	Santa Cruz Biotechnology
Ki-67	Primary	1:100	Santa Cruz Biotechnology
Flk	Primary	1:100	Cell Signaling Technologies
E-cadherin	Primary	1:100	BD Bioscience
Caspase 3	Primary	1:200	Cell Signaling Technologies
Cy3	Secondary	1:200	Jackson ImmunoResearch
Cy5	Secondary	1:200	Jackson ImmunoResearch

Lysozyme was quantified by percentage of immunofluorescent-positive cells per hemi-crypt by a trained, blinded observer with ImageJ. Ki-67-positive cells were counted in the crypt and amplifying zone by the number of positive cells per position by a trained, blinded observer with ImageJ.

### Confocal Microscopy and Three-Dimensional Volume Reconstruction

VEGF mutant and littermate duodenal cross sections were prepared as described above and imaged for FITC and DAPI immunofluorescence using a Zeiss LSM 710 confocal microscope. Sixty micron sections were imaged at an individual z stack thickness of 1 μm to allow for accurate three-dimensional volume reconstruction using IMARIS software. Confocal images were imported into IMARIS version 7.7.2 and volume reconstruction analysis was performed using region of interest selection of FITC-labeled vasculature of individual hemivilli. Threshold values were obtained from littermate controls using autothresholding algorithms and these values were applied to VEGF mutants to allow for proper comparison of three-dimensional volume analysis. Sixty villi in total from four individual mice per group were analyzed.

### VEGF Protein Quantification

Quantification of VEGF protein was executed via enzyme-linked immunosorbent assay (ELISA) on frozen tissue sections for the VEGF mutants and littermate controls or serum from VEGF mutant OU culture. Flash frozen duodenal sections stored at -80°C were selected. The Bradford protein assay method was employed to determine protein concentration. The frozen tissue was homogenized in 200 μL of extraction buffer (1M Tris-HCl, 5M NaCl, 10% Triton, 10% NaDeoxycholate, 0.5M EDTA, 1% PMSF, 10% phosphatase inhibitor, 1% protease inhibitor and distilled water). The homogenized tissue was centrifuged at 10,000 rpm for 10 minutes at 4°C and the supernatant was transferred to a new 1.5 mL eppendorf tube. The Bradford reagent was diluted as recommended by the manufacturer. The bovine serum albumin protein standards (BioRad; Cat# 500–02) and the samples were prepared in duplicates. These were placed in disposable cuvettes and absorbance was measured at a wavelength of 595nm in a spectrophotometer (Pharmacia Ultrospec III). A standard curve was prepared and the concentrations of the total protein were determined. Mouse VEGF ELISA was performed in duplicates with the suggested protocol of the manufacturer (Signosis; EA-2401). The absorbance for the wells were read at 450 nm (PerkinElmer; Cat# Victor^2^ 1420) and a standard curve was plotted to find the concentration of VEGF present in the tissue. The total VEGF protein concentration (pg/mL) was divided by the total protein concentration (μg/mL) to determine the amount of VEGF present per mg of total protein (pg/mg) in tissue samples. VEGF protein concentration in OU cultures was expressed as ng/ul.

### Reverse Transcription Quantitative Polymerase Chain Reaction

The duodenal or OU samples placed in RNAlater and stored in -80°C were thawed for RNA extraction. Tissue homogenization was performed with a rotor-stator homogenizer (Qiagen; TissueRuptor). RNA was extracted with the RNeasy Mini Kit according to the manufacturer protocol (Qiagen; Cat# 74104) with 350 μL as the appropriate volume. The concentration and purity of RNA was determined with a microvolume spectrophotometer (NanoDrop 2000; Thermo Scientific). All samples had a 260/280 ratio of 2.0 or higher (Mean 2.1 ± 0.01), indicating high purity of the isolated RNA. Reverse transcription was performed with the cDNA kit (BIO-RAD; Cat# 170–8896) and the mixture contained 1 μl RNA in a 20 μl reaction volume. The thermo cycler (BIO-RAD; Cat#C1000) temperatures were set according to the manufacturer’s recommendations.

VEGF transcript overexpression was confirmed in VEGF mutants with RT-qPCR. SYBR green was performed for the transgenic VEGF mutants. The cDNA for the VEGF mutants was employed in this reaction. The reaction contained 7.5 μl of SYBR Green I Master Mix (Roche; Cat# 0707516001), 0.2 μl Tet(o)-VEGF forward and reverse primers (Table 1), 6.1 μl RNase-free water and 1 μl of cDNA. The Roche Lightcycler 480 performed RT-qPCR with 1 cycle at 95°C for 10 minutes, 45 cycles at 94°C for 1 minute followed by 55°C for 1 minute and 72°C for 1 minute each, and 1 cycle at 40°C for 30 seconds. The reactions were performed in triplicate and single outliers were removed for quantification. Roche Lightcycler 480 was used to calculate the relative gene expression of Tet(o)-VEGF related to GAPDH. sFlt-1 transcript was confirmed in sFlt-1 mutants in a similar fashion.

Quantitative PCR was performed with the resulting cDNA in the Roche LightCycler 480 system with hydrolysis probes (Table 3) in a multiplex reaction with GAPDH as a reference gene. Each reaction contained 0.2 μl GAPDH primers (Roche; Cat# 05046211001), 0.2 μl GAPDH probe (Roche; Cat# 05046211001), 0.2 μl 100 μM left primer, 0.2μl 100 μM right primer, 0.2 μl hydrolysis probe (Table 3), 7.5 μl master mix (Roche; Cat#04707494001), 5.5 μl of RNase free water and 1 μl cDNA. Gene expression of Bmi1, Lgr5, Atoh1 and Hes1 (n = 6), as well as of Sox9, DII1, Wdr43, EphB2 and Bmp4 (n = 6) was analyzed for VEGF and mutants. Similarly, gene expression of Bmi1, Lgr5, Atoh1 and Hes1 (n = 6) and Sox9, DII1, Wdr43, EphB2 and Bmp4 (n = 6) was quantitated in sFlt-1 mutants. To investigate the effect of VEGF overexpression and suppression on angiogenesis in the postnatal mouse duodenum, quantitative RT-qPCR was performed for VE-cadherin, a marker of endothelial-specific cell-cell adherence junctions (Table 3). The reactions were performed in clear, 96-well plates (Roche; Cat# 05102413001) under the following conditions: 1 cycle at 95°C for 10 minutes, 45 cycles at 95°C for 10 seconds followed by 60°C for 30 seconds and 72°C for 1 second each, and 1 cycle at 40°C for 30 seconds. Each PCR reaction was run in triplicate and single outliers that occurred in the technical replicates were removed for quantification. Roche Lightcycler 480 was used to calculate the relative gene expression of the target gene over the reference gene with advanced relative quantification calculations.

**Table 3:** Primers and Hydrolysis Probes Used for RT-qPCR.

Primers	Sequence
VEGF-A F	5’-ACTGGACCCTGGCTTTACTG-3’
VEGF-A R	5’-TCTGCTCTCCTTCTGTCGTG-3’
sFlt-1 F	5’-CGACTCACTATAGGGAGACCC-3’
sFlt-1 R	5’-TGGCCTGCTTGCATGATGTGCTGG-3’
Bmi1 F	5’-CAAAACCAGACCACTCCTGAA-3’
Bmi1 R	5’-CCATGATAGGCTTTGATGACTTT-3’
Lgr5 F	5’-CTTCACTCGGTGCAGTGCT-3’
Lgr5 R	5’-CAGCCAGCTACCAAATAGGTG-3’
Atoh1 F	5’-TGCGATCTCCGAGTGAGAG-3’
Atoh1 R	5’-CTCTTCTGCAAGGTCTGATTTTT-3’
Hes1 F	5’-TGCCAGCTGATATAATGGAGAA-3’
Hes1 R	5’-CCATGATAGGCTTTGATGACTTT-3’
Sox9 F	5’-GAAAGACCACCCCGATTACA-3’
Sox9 R	5’-TCCGCTTGTCCGTTCTTC-3’
DII1 F	5’-ACATGTTCCTGCCGACCT-3’
DII1 R	5’-GGGCTAGGAGCACACTCATC-3’
Wdr43 F	5’-GGACAGAAGGCAAAATTGGT-3’
Wdr43 R	5’-TCTCTGGAACCTCATCGTCA-3’
EphB2 F	5’-TATGCCGCAACGGCTACT-3’
EphB2 R	5’-GTCTCGTTGACGCTGGAGAT-3’
Bmp4 F	5’-GAGGAGTTTCCATCACGAAGA-3’
Bmp4 R	5’-GCTCTGCCGAGGAGATCA-3’
VEcad F	5’-GTTCAAGTTTGCCCTGAAGAA-3’
VEcad R	5’-GTGATGTTGGCGGTGTTGT-3’

The above are the nucleotide sequences for genes from RT-qPCR and the appropriate hydrolysis probes from the Roche Applied Science Universal Probe Library that were employed in the RT-qPCR reactions.

### Intravascular-Immunofluorescence Labeling by Intracardiac FITC-Dextran Injections

Mice were anesthetized using standard IACUC procedures. Once fully anesthetized, mice were placed supine with arms and legs extended. Silk tape was placed across the abdomen and along the upper extremities to secure the animal firmly on the working surface. The chest was shaved to allow for visualization of the rib cage. The mice were then prepped and draped in a sterile fashion. A 2mg/ml FITC-dextran (Sigma FD2000S) solution was made in PBS and protected from light. Using a 1ml insulin syringe with 26-G needle, 200 μl of air was drawn up into the barrel followed by 200 μl of FITC-dextran solution. The needle was inserted into the second intercostal space 3 mm to the left of the sternum, directing the tip into the center of the chest (45° to the right, 45° to the horizontal plane and pointing towards the right flank of the mouse) to a depth of 6 mm. Pulsatile flow of red blood into the hub of the needle indicated correct placement of the needle into the left ventricle and 200 μl of FITC-dextran solution was injected per mouse while avoiding the introduction of air bubbles into the circulatory system. The needle was quickly withdrawn and pressure placed on the chest with alcohol wipes for 1 minute to prevent extravasation into the chest. Mice were sacrificed 3 minutes after FITC-dextran injection and intestinal tissue immediately harvested and fixed in 4% paraformaldehyde.

### Mouse Enteroid Cultures

We isolated mouse enteroids from VEGF mutant mice using a previously establish protocol [20]. Briefly, crypts were released from murine small intestine by incubation for 30 min at 4°C in PBS containing 2 mM EDTA. Isolated crypts were counted and a total of 500 crypts were mixed with 50 μl of Matrigel (BD Bioscience) and plated in 24-well plates. After Matrigel polymerization occured, 500 μl of crypt culture medium (Advanced DMEM/F12 (Invitrogen) containing growth factors (10–50 ng/ml EGF (Peprotech), 500 ng/ml R-spondin 1 (R&D systems) and 100 ng/ml Noggin (Peprotech)) was added. For sorting experiments, isolated crypts were incubated in culture medium for 45 min at 37°C, followed by trituration with a glass pipette. Dissociated cells were passed through a cell strainer with a pore size of 20 μm. Growth medium was changed every 4 days.

### Mouse Organoid Units

The VEGF mutant duodenal samples were put into a 10 cm Petri-Dish with cold Hanks’ balanced salt solution (Life Technologies, Inc.) to clean the luminal contents. The tissue is then cut into 2x2 mm sections in the Petri-Dish. The tissue is placed in a 50 mm centrifuge tube and centrifuged for 1 minute at 8000 rpm. The tissue is washed 3 times with cold Hanks’ salt solution, centrifuging between washes and removing the supernatant. The tissues were then digested with 800 μg/mL collagenase (Worthington) and 0.12 mg/mL dispase (Invitrogen) for 20 minutes. The digestion was stopped by adding high glucose Dulbecco’s Modified Eagle Medium (DMEM) with 4% Sorbitol (Sigma, Inc.) and 10% fetal bovine serum (FBS) (Invitrogen). The digestion was triturated with a 10 mL pipette for approximately 10 minutes. Large debris sediment was removed by centrifugation and the supernatant was transferred to a 50 mL Falcon tube. The mixture was centrifuged at 500 rpm for 10 minutes and the supernatant was discarded. The pellet was resuspended in 2 mL high glucose DMEM with 10% FBS. This solution was mixed with Matrigel (BD Bioscience) at a ratio of 1:1. 100 μL of the Matrigel-organoid unit (OU) mixture was placed in each well of a 6-well plate. The plate was incubated at 37°C for 30 minutes. 2 mL DMEM was then added to each well. The cultures were incubated overnight at 37°C with 5% CO_2_ and medium was changed on days 1, 3, 5, 7 and 9. Experimental OU had 2 μL of doxycycline added to the medium, while controls did not. The OU were imaged on days 1, 3, 5, 7, and 9. On day 10, each well was scraped and individually collected and placed in a 1.5 mL Eppendorf tube. These tubes were centrigued at 1000 rpm for 1 minute. The media was then removed and 200 μL of RNAlater was added to the Eppendorf tube. These were stored at 4°C until RNA extraction was performed as described above. Diameter of OU were measured on ImageJ by a trained, blinded observer.

## Results

### VEGF overexpression resulted in elevated levels of VEGF mRNA and protein. sFlt-1 overexpression resulted in an increased expression of sFlt-1 in enteroid culture and negative feedback reduction of VEGFR1 receptor in duodenum

**Fig 1:**
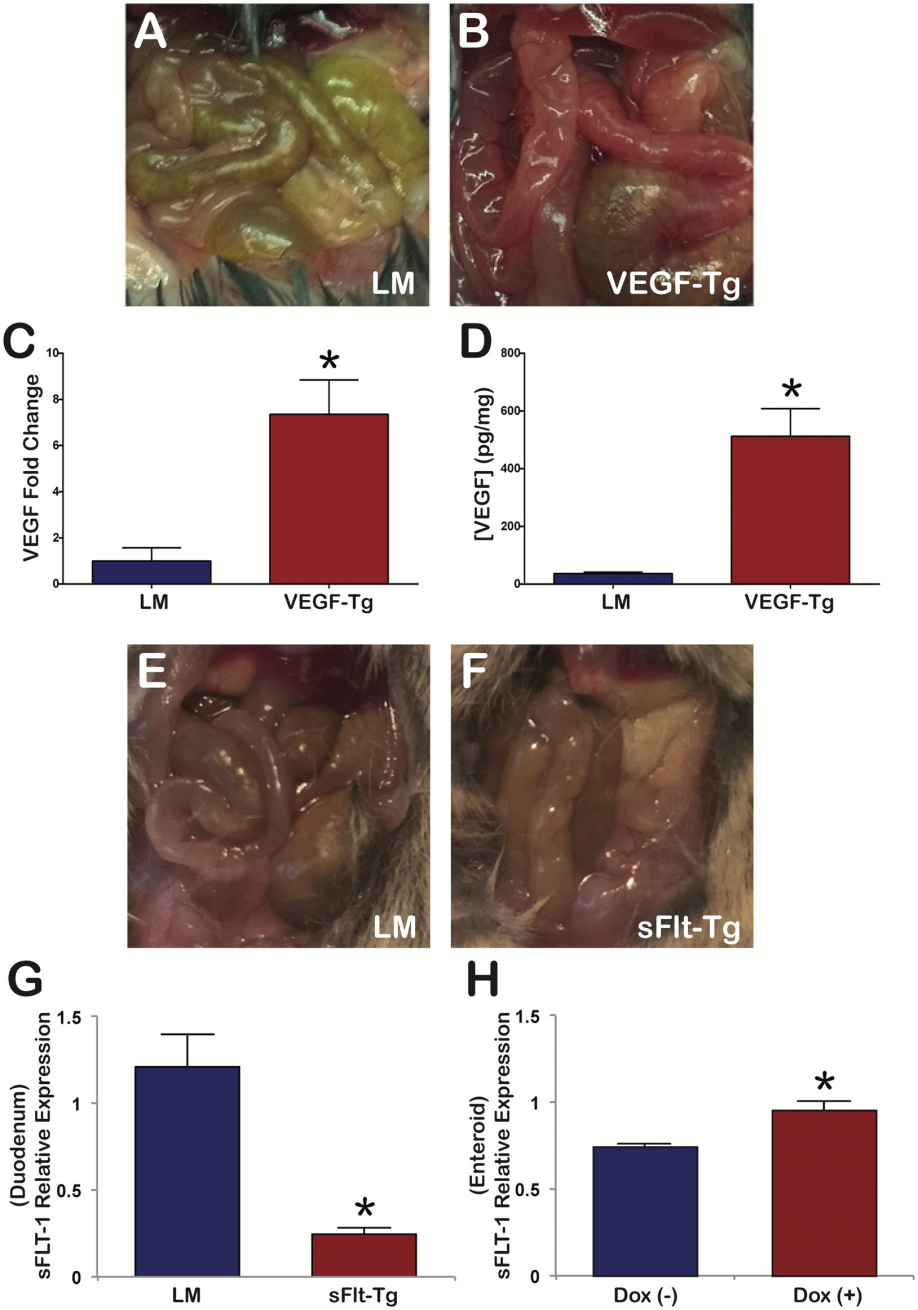
VEGF and sFlt-1 mutant phenotypes. VEGF mutant overexpression of VEGF by RT-PCR and ELISA. sFlt-1 mutant expression of sFlt-1 by RT-PCR. (A) VEGF littermate mice revealed a normal, pink to yellow phenotype of the intestine. (B) VEGF mutants demonstrated a deeper red color of the intestine. (C) VEGF mutant mice revealed a 7.36 ± 1.49 SEM-fold increase of transgenic VEGF transcript compared to littermates. (D) VEGF mutant mice duodenum displayed a 13.92 ± 2.60 SEM-fold increase of VEGF protein compared to the littermates; N = 3 mice per group; *p<0.05; Error bars = SEM. (E) s-Flt-1 littermate mice with a normal intestine. (F) sFlt-1 mutants demonstrated paler coloration of the intestine. (G) sFlt-1 mutant mice duodenum demonstrated a significant decrease in full length Flt-1 and sFlt-1 transcript at 21 days compared to littermates, suggesting a negative feedback response. N = 3 mice per group; *p<0.001; Error bars = SEM. (H) sFlt-1 mutant small intestine enteroid cultures demonstrated increased expression of full length Flt-1 and sFlt transcript after 3 days of doxycycline administration. N = 25 OU per well; 6 wells; *p = 0.019; Error bars = SEM.

Following 21 days of doxycycline induction, triple transgenic VEGF mutants (VEGF-Tg) were confirmed by demonstrating overexpression of VEGF in the postnatal mutant duodenum by RT-qPCR (Fig 1C) and ELISA (Fig 1D). VEGF mutant mice displayed a 7.36 ± 1.49 SEM-fold increase in the transgenic VEGF transcript (p = 0.02) compared to littermates. Similarly, VEGF protein concentration was significantly higher in VEGF mutant duodenum (512.51 ± 95.69 pg/mg SEM) compared to littermates (36.81 ± 4.54 pg/mg SEM) (Fig 1D). This represents a 13.92 ± 2.60 SEM-fold change (p = 0.008).

Following 21 days of doxycycline induction, the triple transgenic sFlt-1 mutants (sFlt-Tg) were confirmed by demonstrating expression of sFlt-1 in the postnatal mutant duodenum and epithelial enteroid culture by RT-qPCR (Fig 1G and 1H). At 21 days of doxycycline treatment, sFlt-1 mutants had significantly decreased sFlt-1 expression compared to littermates (1.21 ± 0.19 SEM versus 0.24 ± 0.04 SEM; p<0.001) (Fig 1G). RT-PCR and ELISA of VEGF-A in the duodenum failed to demonstrate a significant decrease between sFlt-1 mutant duodenum (0.90 ± 0.09 SEM; p = 0.6 and -0.117 pg/mg ± 0.004 SEM; p = 0.88, respectively) versus littermates (0.99 ± 0.14 SEM and -0.123 pg/mg ± 0.003 SEM, respectively) (S1G and S1H Fig). However, sFlt-1 mutant enteroid cultures demonstrated a significant increase in sFlt-1 expression upon doxycycline treatment compared to controls (0.958 ± 0.02 SEM versus 0.746 ± 0.09 SEM; p = 0.019) (Fig 1H). Doxycycline addition in sFlt-1 enteroid culture did not alter the expression of another VEGF receptor, Flk (0.156 ± 0.02 SEM versus 0.152 ± 0.02 SEM; p = 0.85) and did not promote endothelial cell growth in culture as enteroid cultures are devoid of endothelium (S2A and S2B Fig).

### VEGF overexpression resulted in increased villus angiogenesis and taller villi. Reduced VEGF bioavailability produced a shorter, paler intestine with a diminutive cecum and smaller villi with longer, but fewer crypts per measured length

The gross appearances of the duodenum in VEGF mutants and littermates were distinct. Littermate duodenum was a pink to yellow color (Fig 1A) compared to a deep red intestine seen in VEGF mutant mice (Fig 1B). Compared to littermates, sFlt-1 mutant mice were smaller and had decreased body mass (p<0.001), as well as a swollen anus (S1A Fig). The duodenum of sFlt-1 mutant mice was paler in comparison to littermates (Fig 1F and 1E, respectively). The length of the intestine was also shorter with a diminutive cecum in sFlt-1 mutant mice (S1B Fig). The cecum of sFlt-1 mutant mice demonstrated decreased number of goblet cells compared to littermates (S1F and S1E Fig, respectively).

**Fig 2:**
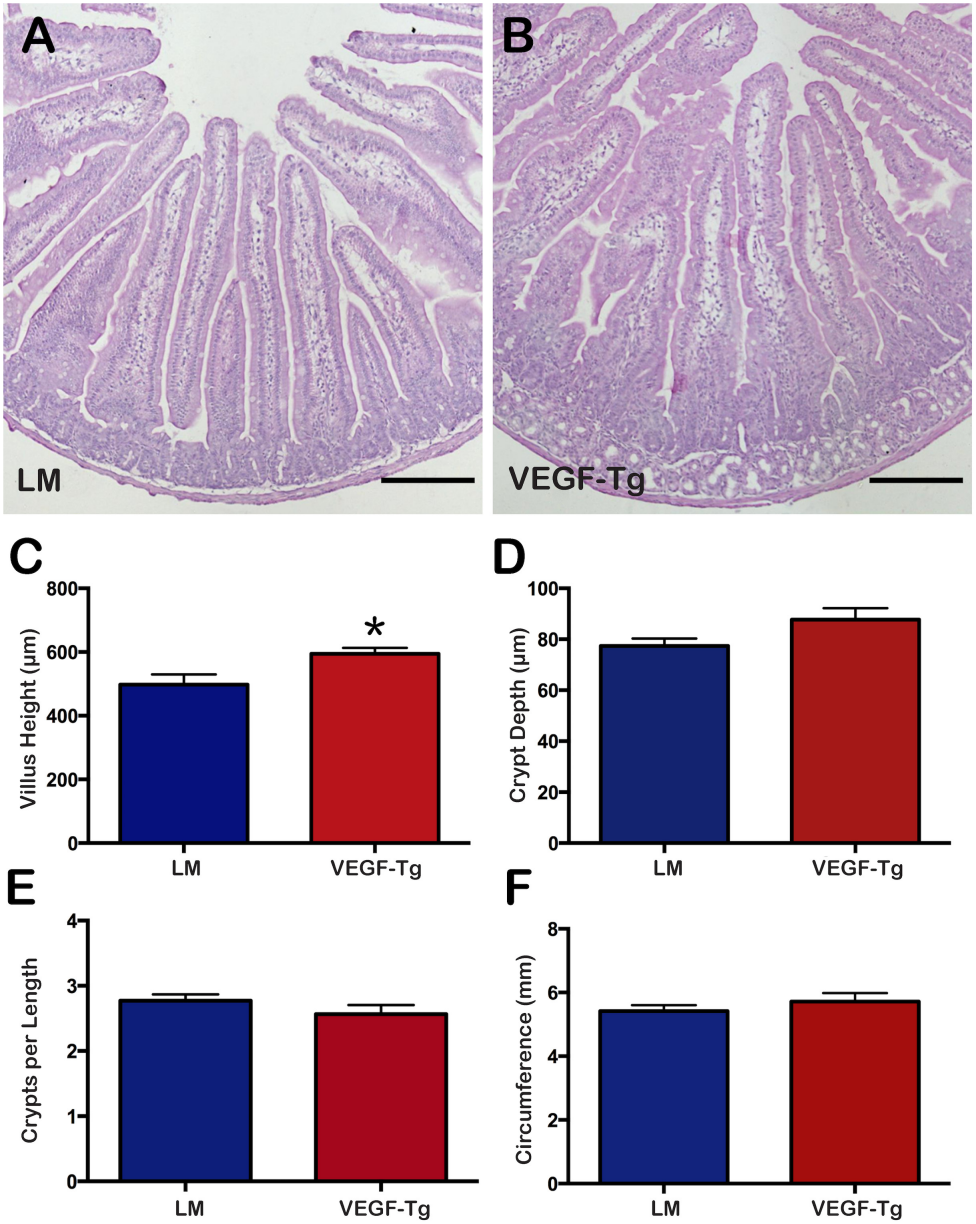
VEGF mutant duodenum displayed longer villi compared to littermates. (A) Cross-section of littermate duodenum. (B) Cross-section of VEGF mutant duodenum. Scale bars = 200 μm. (C) VEGF mutant mice had significantly taller villus height compared to littermates. (D) There was no significant difference in crypt depth between VEGF mutants and littermates. (E) There was no significant difference in the number of crypts per measured length between VEGF mutants and littermates. (F) There was no significant difference in duodenal circumference between VEGF mutants and littermates. N = 6 mice per group. *p<0.05; Error bars = SEM.

Histological sections by H&E demonstrated significantly higher villus height in VEGF mutant duodenum (Fig 2B) at 594.1 ± 19.03 μm SEM compared to littermates (Fig 2A) at 497.4 ± 32.00 μm SEM (p = 0.03) (Fig 2C). There was no significant difference in the crypt depth (87.70 ± 4.47 μm SEM in VEGF mutants versus 77.41 ± 2.92 μm SEM in littermates; p = 0.08) (Fig 2D) or in the number of crypts per length measured in VEGF mutants compared to littermates (2.56 ± 0.14 μm SEM versus 2.77 ± 0.09 μm SEM, respectively; p = 0.3) (Fig 2E). No significant difference in duodenal circumference existed between VEGF mutants and littermates (5.72 ± 0.27 mm SEM versus 5.41 ± 0.19 mm SEM, respectively; p = 0.4) (Fig 2F).

**Fig 3:**
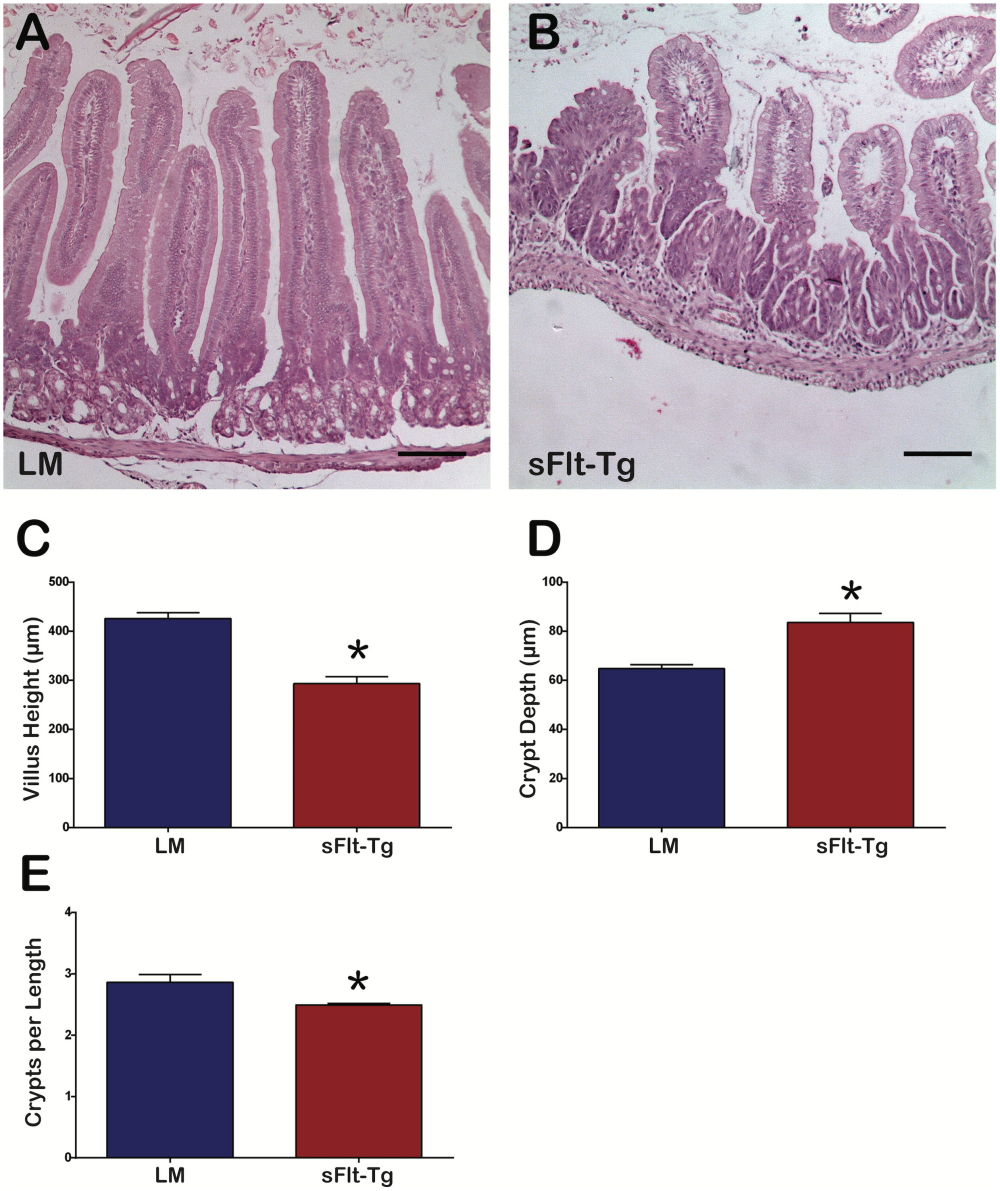
sFlt-1 mutant duodenum displayed shorter villi, increased crypt depth and fewer crypts per length measured. (A) Cross-section of littermate duodenum. (B) Cross-section of sFlt-1 mutant duodenum; Scale bars = 100 μm (C) sFlt-1 mutant mice had shorter villi compared to littermates (*p<0.0001). (D) sFlt-1 mutant mice had deeper crypts compared to littermates (*p<0.0001). (E) sFlt-1 mice had fewer crypts per measured length (*p = 0.03); N = 6 mice per group; Error bars = SEM.

Duodenum of sFlt-1 mutants (Fig 3B) displayed shorter villi compared to littermates (Fig 3A) (293.3 ± 14.4 μm SEM versus 426.0 ± 11.7 μm SEM, respectively; p<0.0001) (Fig 3C) and taller crypt depth (83.6 ± 3.7 μm SEM versus 64.7 ± 1.7 μm, respectively; p<0.0001) (Fig 3D). There were also significantly fewer crypts per length measured in sFlt-1 mutants compared to littermates (2.49 ± 0.02 μm SEM versus 2.86 ± 0.12 μm SEM, respectively; p = 0.03) (Fig 3E).

### VEGF augmentation in intestinal epithelium leads to increased mucosal angiogenesis and vascular permeability

**Fig 4:**
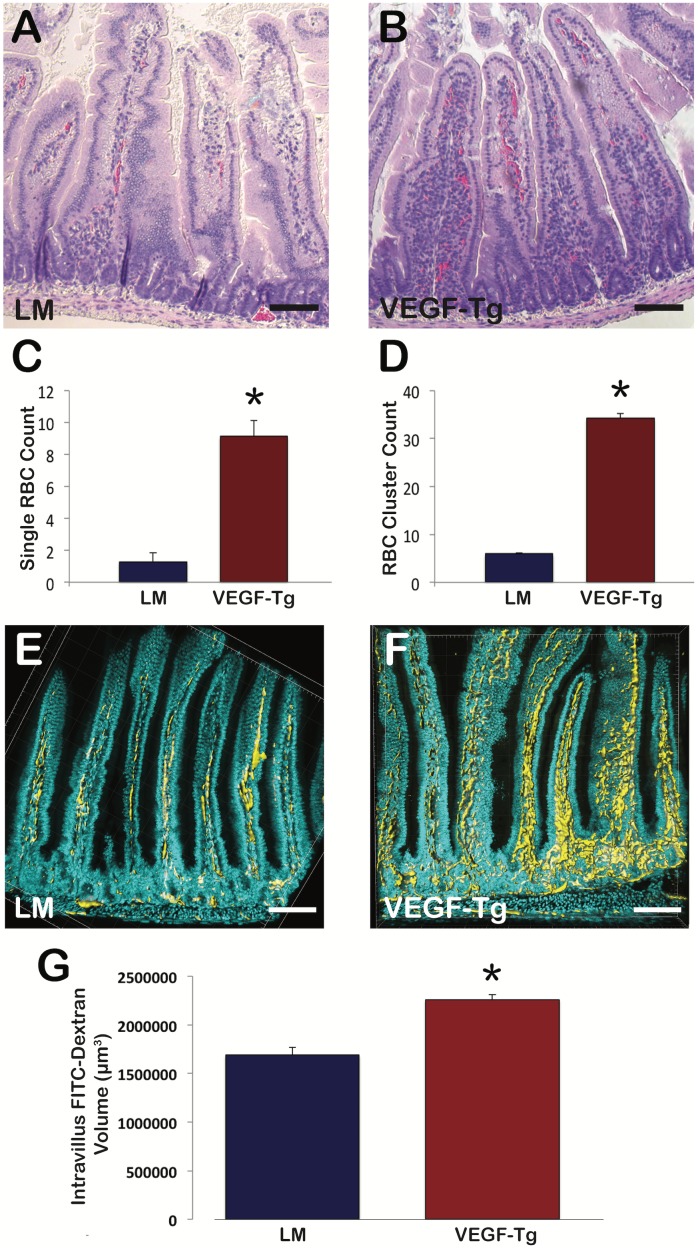
VEGF mutant villi demonstrated increased angiogenesis and vascular permeability. **(A)** Cross-section of littermate duodenum. (B) Cross-section of VEGF mutant duodenum; Scale bars = 100 μm (C) Single RBC counts per individual villus were increased in VEGF mutant mice; N = 3 mice per group; 20–35 villi per mouse were evaluated; *p<0.001; Error bars = SEM. (D) RBCs cluster counts (>3 RBCs in close contact) per individual villus were increased in VEGF mutant mice; N = 3 mice per group; 20–35 villi per mouse were evaluated; *p<0.001; Error bars = SEM. (E) Three-dimensional cross-section of littermate duodenum. (F) Three-dimensional cross section of VEGF mutant duodenum. Nuclei labeled with DAPI (Cyan) and vasculature labeled with FITC-dextran (Yellow); Scale bars = 100 μm. (G) VEGF mutant vasculature displays increased volume compared to littermates; N = 4 mice per group; *p<0.001; Error bars = SEM.

H&E histological cross sections demonstrated a significant increase in the amount of single and multiple clusters of RBCs within the villi of VEGF mutant mice compared to littermates (Fig 4B and 4A, respectively). Single RBC counts (34.28 ± 3.24 SEM versus 5.98 ± 0.54 SEM; p<0.001) and multiple RBC clusters (9.13 ± 0.97 SEM versus 1.29 ± 0.21 SEM; p<0.001) per villus were significantly increased in VEGF mutants compared to littermates, suggestive of increased vascular leak and angiogenesis. To further evaluate vascular leak and angiogenesis, intracardiac FITC-dextran injections were performed to label the vasculature and 60 μm cross sections were imaged using confocal microscopy (Fig 4F and 4E, respectively). Three-dimensional volume reconstruction demonstrated significant increase in intravillus FITC-dextran volume in VEGF mutants as compared to littermates (2259000 μm^3^ ± 54200 SEM versus 1689083 μm^3^ ± 75245 SEM; p<0.001) (Fig 4G).

### VE-cadherin expression significantly increased in VEGF mutants and decreased in sFlt-1 mutants

**Fig 5:**
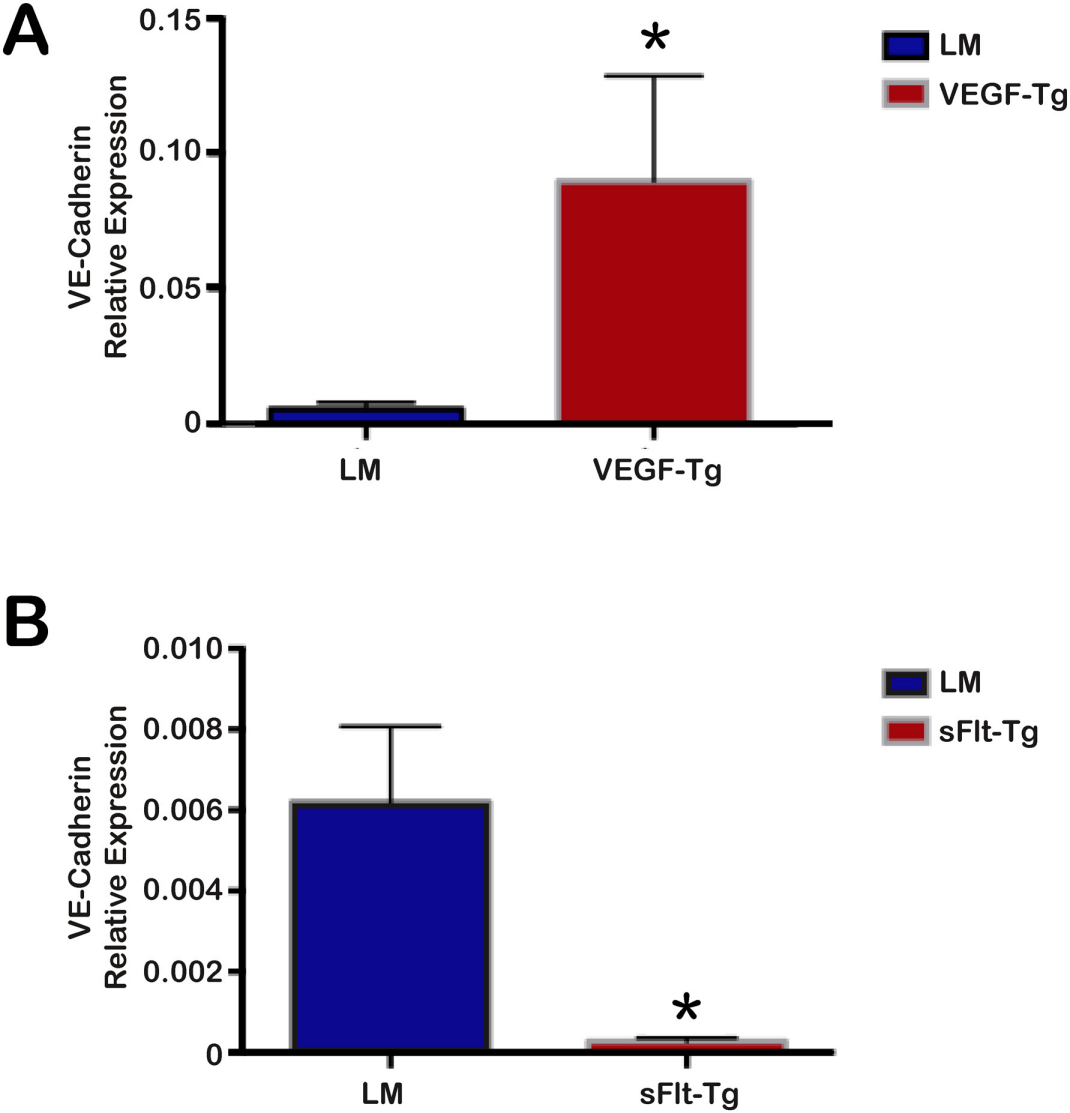
VEGF mutants demonstrated increased VE-cadherin expression compared to littermates, while sFlt-1 mutants demonstrated decreased expression. (A) VEGF mutants demonstrated a 16.5-fold increase in VE-cadherin expression compared to littermates (*p = 0.04). (B) sFlt-1 mutants had a 22.4-fold reduction in VE-cadherin expression compared to littermates (*p = 0.01); N = 3 mice per group; Error bars = SEM.

Given the distinct increase in intravascular volume and vascular leak in the hemivillus of VEGF mutants, we further explored the effects on endothelial cells in VEGF and sFlt-1 mutants by performing RT-qPCR for VE-cadherin, an endothelial cell marker [21]. VEGF mutant mice showed a significant increase in VE-cadherin expression compared to littermates (p = 0.04), with a 16.5-fold increase in expression (Fig 5A). In contrast, the littermates of sFlt-1 mutants demonstrated significantly increased expression of VE-cadherin compared to the sFlt-1 mutant mice (p = 0.01), with a 22.4-fold increase in expression in littermates compared to sFlt-1 mutants (Fig 5B).

### VEGF mutants have increased Ki-67-positive cells per hemivillus extending higher into the transit-amplifying zone, whereas sFlt-1 mutants display Ki-67-positive cells residing lower in the crypt

**Fig 6:**
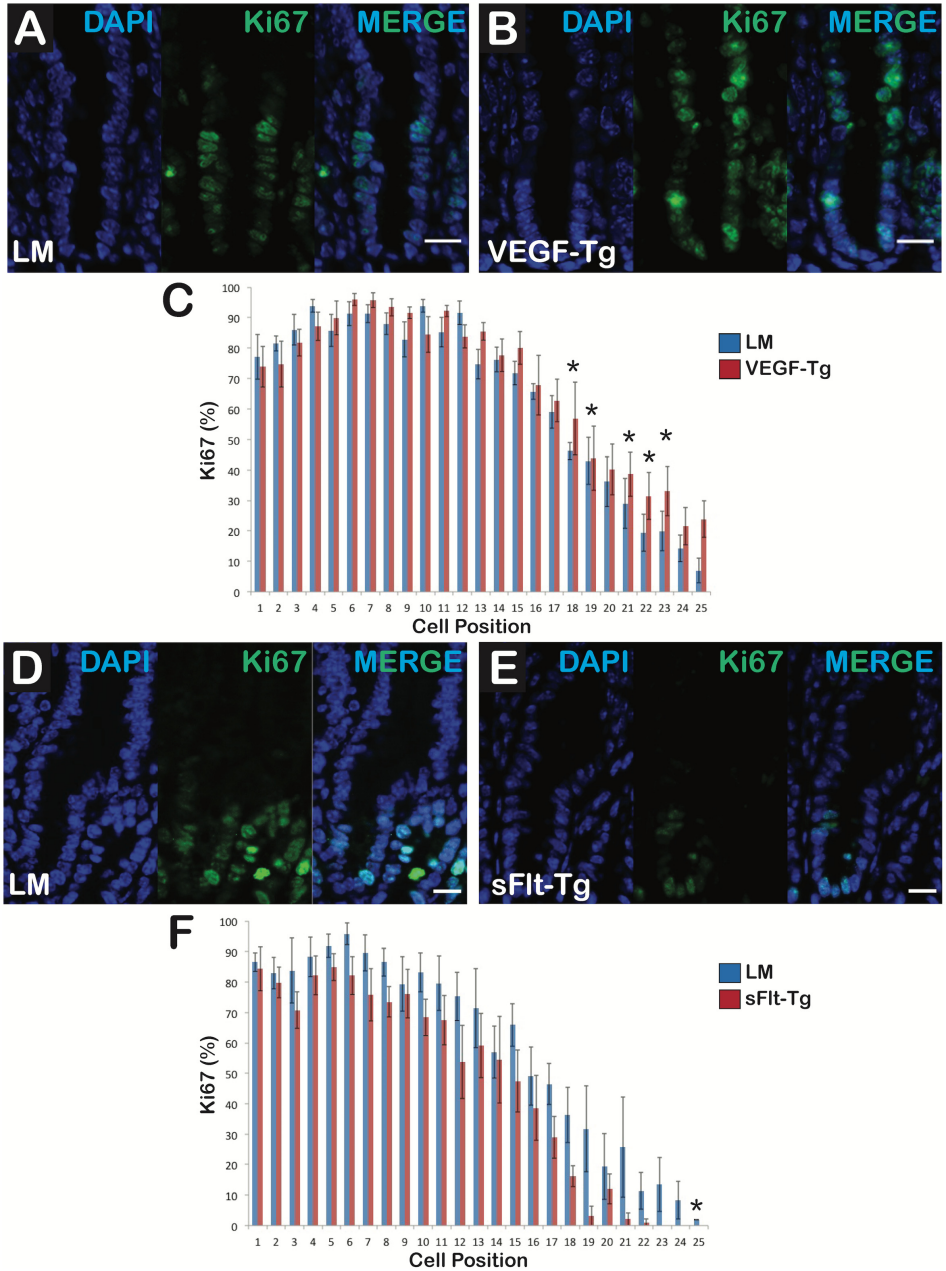
VEGF augmentation increased epithelial cell proliferation in the transit-amplifying zone whereas reduced VEGF bioavailabity restricted proliferation to the crypt bases. (A) Littermate crypts had typical proliferation in the crypt and transit-amplifying zone of the duodenum. (B) VEGF mutant crypts displayed increased proliferation at higher positions in the transit-amplifying zone compared to littermates. First panel shows nuclei with DAPI staining (Blue), second reveals Ki-67-positive cells (Green) and third panel is the composite (MERGE); N = 6 mice per group; Scale bars = 20 μm (C) VEGF mutants had more Ki-67-positive cells at positions 16 through 25 than the littermates. (D) Littermate crypts had typical proliferation in the crypt and transit-amplifying zone. (E) sFlt-1 mutant crypts displayed decreased proliferation in the transit-amplifying zone compared to littermates. First panel shows DAPI staining (Blue), second reveals Ki-67-positive cells (Green) and third is the composite (MERGE); N = 6 mice per group; Scale bars = 10 μm (F) sFlt-1 mutants had fewer Ki-67-positive cells at positions 10 through 25 than littermates. N = 6 mice per group; *p<0.05; Error bars = SEM.

Duodenal sections of VEGF mutants (Fig 6B) displayed more Ki-67-positive cells per hemivillus compared to littermates (Fig 6A). In the VEGF mutants, the Ki-67-positive cells extended to a higher position in the transit-amplifying zone compared to littermates (Fig 6C). sFlt-1 mutants (Fig 6E) exhibited fewer Ki-67-positive cells per hemivillus compared to littermates (Fig 6D). Ki-67-positive cells were identified lower in the crypts of sFlt-1 mutants compared to littermates (Fig 6F). Caspase 3 staining revealed no significant difference between VEGF mutants, sFlt-1 mutants and their comparable littermates (S3 Fig).

### sFlt-1 mutants demonstrated an increase in lysozyme-positive Paneth cell population within the intestinal crypts

**Fig 7:**
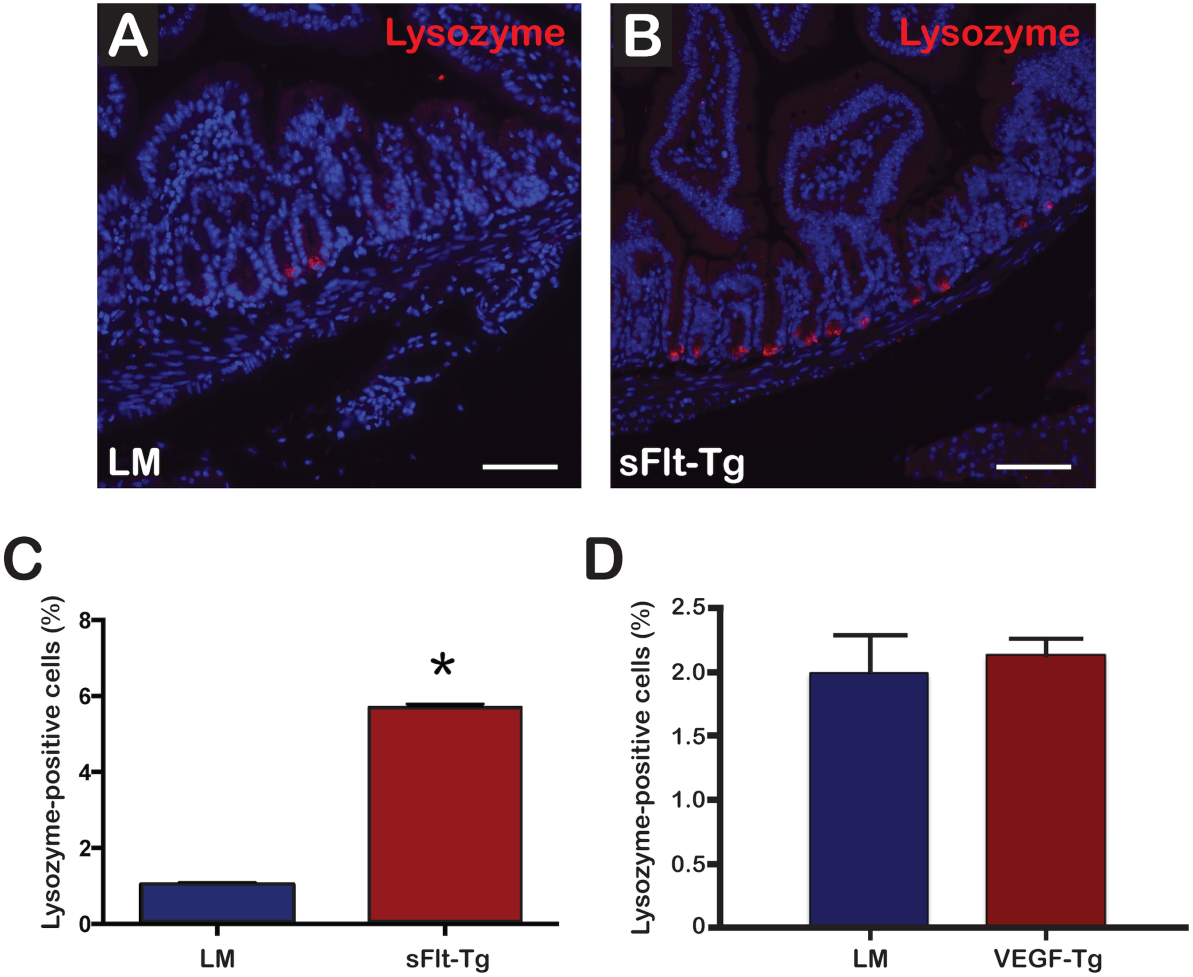
sFlt-1 mutant mice demonstrated increased lysozyme-positive cells per hemicrypt. (A) Immunofluorescence demonstrating lysozyme (Red) staining in littermates. (B) Lysozyme staining in sFlt-1 mutants showing increased staining in the duodenal crypts; Scale bars = 75μm. (C) The average percentage of lysozyme-positive cells per hemicrypt was significantly increased in sFlt-1 mutants (*p = 0.005). (D) No significant difference in the average percentage of lysozyme-positive cells per hemicrypt was appreciated in VEGF mutants; N = 4 mice per group; Error bars = SEM.

Immunofluorescence of duodenal sections revealed significantly more lysozyme-positive cells per hemicrypt in sFlt-1 mutants compared to littermates (Fig 7A and 7B). The percentage of lysozyme-positive cells per hemicrypt was 5.70 ± 0.70% SEM for sFlt-1 mutants (Fig 7C) compared to 1.036 ± 0.05% SEM (p = 0.0005) for littermates (Fig 7C). No significant difference in lysozyme staining was appreciated in VEGF mutants compared to littermates (2.14 ± 0.11% SEM versus 1.99 ± 0.29% SEM; p = 0.48) (Fig 7D).

### VEGF mutants demonstrate a significant decrease in expression of the intestinal stem cell marker Lgr5. sFlt-1 mutants demonstrate reduced Bmi1 and Wdr43 expression and a concomitant increase in EphB2 and Sox9 expression

RT-qPCR was performed for stem/progenitor and early differentiation markers in VEGF mutant and sFlt-1 mutant mice. These genes included Bmi1, Lgr5, Atoh1, Hes1, Sox9, DII1, Wdr43, EphB2, and Bmp4. Bmi1 is a marker for slow cycling intestinal stem cells, typically located at the +4 position [22]. Lgr5, or leucine-rich repeat-containing G-protein coupled receptor 5, is a marker for rapid cycling intestinal stem cells, also known as crypt base columnar cells (CBCs)[23]. Atoh1 marks progenitor cells of the secretory lineage, while Hes1 is expressed in progenitor cells of the absorptive lineage [24]. Dll1 is a marker for secretory precursors [25]. Sox9 marks CBCs when expressed at low levels or Paneth cells when expressed at high levels [26]. Wdr43 is a marker for transit-amplifying cells [23]. EphB2 marks differentiating cells at low levels of expression and intestinal stem cells and progenitor cells at high levels of expression [27]. Bmp4 inhibits de novo crypt formation and is expressed in the intravillus mesenchyme [28].

**Fig 8:**
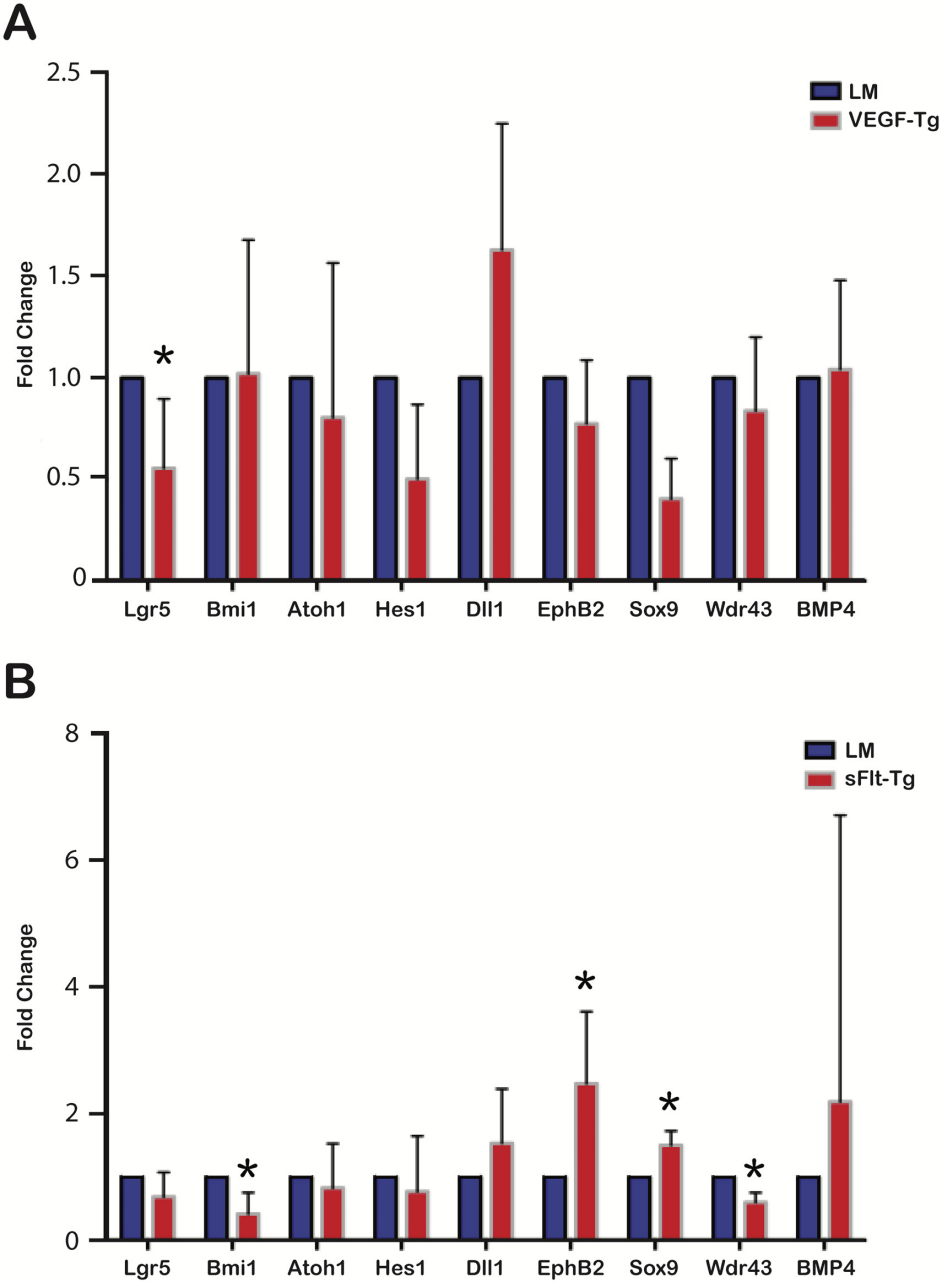
VEGF and sFlt-1 mutants exhibit changes in stem cell marker gene expression by RT-qPCR. (A) VEGF mutants demonstrated 0.55-fold reduction in Lgr5 expression compared to littermates (*p = 0.04). There were no other significant differences in stem cell marker expression in the VEGF mutants compared to littermates; N = 6 mice per group; Error bars = SEM. (B) Bmi1 expression decreased 0.42 fold (*p = 0.03) and Wdr43 expression decreased 0.60 fold (*p = 0.004) in sFlt-1 mutants compared to littermates. sFlt-1 mutants demonstrated a 2.47-fold increase in expression of EphB2 (*p = 0.02) and a 1.50-fold increase in Sox9 expression (*p = 0.004); N = 6 mice per group; Error bars = STDEV.

VEGF mutants demonstrated a 0.55 ± 0.12 SEM-fold reduction in Lgr5 expression compared to littermates (p = 0.04) (Fig 8A). There was no significant change in other stem/progenitor cell marker gene expression in VEGF mutants compared to littermates. sFlt-1 mutants exhibited a 0.42 ± 0.19 SEM-fold reduction in Bmi1 expression (p = 0.03) and a 0.60 ± 0.09 SEM-fold reduction in Wdr43 expression (p = 0.004) compared to littermates. EphB2 and Sox9 expression were increased 2.47 ± 0.24 SEM fold (p = 0.02) and 1.50 ± 0.09 SEM fold (p = 0.004) respectively in sFlt-1 mutants compared to littermates (Fig 8B).

### VEGF mutant organoid units (OU) exposed to doxycycline are significantly larger than untreated controls. VEGF augmentation in murine OU increases expression of stem/progenitor cell markers Bmi1 and Atoh1 and decreases EphB2 expression

**Fig 9:**
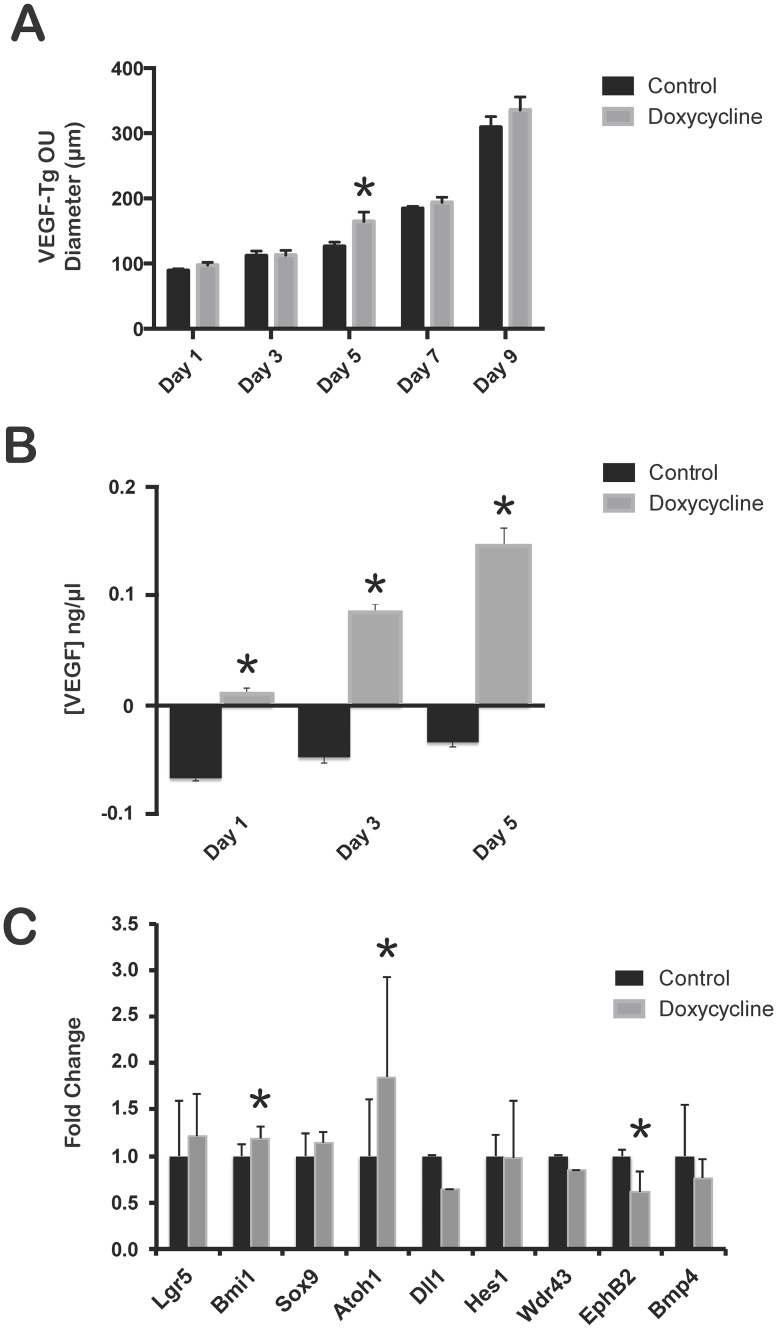
VEGF overexpression in OU culture increased OU size and altered stem/progenitor cell gene expression. (A) The diameter of VEGF mutant OU were measured every other day during a 10-day culture. The diameter of all OU increased over time; however, VEGF mutant OU treated with doxycycline were larger on day 5 compared to controls (*p = 0.04). N = 25 OU per well, 6 wells; Error bars = SEM. (B) VEGF mutant OU exposed to doxycycline demonstrated significant increase in serum VEGF levels over 5 days in culture (*p<0.05). N = 3; Error bars = SEM. (C) Significant increase in Bmi1 and Atoh1 expression and decrease in EphB2 expression was observed in doxycycline-treated VEGF OU compared to controls at 5 days (*p<0.05). N = 3; Error bars = STDEV.

To further isolate the effect of VEGF on the epithelium, we generated OU cultures from intestinal tissue and induced VEGF overexpression by exposure to doxycycline. OU contain both epithelium and mesenchyme, but lack a vascular supply [29–31]. To evaluate the effect of VEGF overexpression on OU *in vitro*, VEGF mutant duodenum OU were cultured and the diameters were measured every other day for 10 days before tissue harvest. OU treated with doxycycline or medium alone grew over the 10-day period. The doxycycline-treated OU were significantly larger on day 5 compared to control (164.36 ± 14.43 μm versus 126.11 ± 6.57 μm; p = 0.04) (Fig 9A). This difference did not persist after day 5. These findings correlated with a temporal increase in VEGF protein expression by ELISA over a period of 5 days in culture (Fig 9B). Doxycycline treated OUs from C57/B6 mice demonstrated no significant size difference over 10 days (S2C Fig). After 5 and 10 days of *in vitro* culture with or without doxycycline, expression of stem cell markers was evaluated in VEGF mutant OU. At 5 days, a significant increase in Bmi1 (1.14 ± 0.13 versus 0.96 ± 0.13; p = 0.03) and Atoh1 (2.54 ± 1.07 versus 1.38 ± 0.60; p = 0.04) expression and decrease in EphB2 (0.68 ± 0.22 versus 1.11 ± 0.07; p = 0.001) expression was observed in doxycycline-treated VEGF mutant OU compared to controls (Fig 9C). No significant difference in the expression of Lgr5, Bmi1, Sox9, Atoh1, Dll 1, Hes1, Wdr43, EphB2, or BMP4 was identified between doxycycline-treated VEGF mutant OU compared to controls at 10 days (S2D Fig).

## Discussion

VEGF overexpression and reduced bioavailability had distinct effects on postnatal small intestine in a murine model. VEGF is excreted in breast milk and decreased in the intestines of formula-fed murine and human neonates that succumb to necrotizing enterocolitis [32–34]. Mesenchymal-driven sFlt-1 mice demonstrate significant changes in body and organ size at 21 days [35]. Given these findings and the dramatic phenotype demonstrated in the Villin-Cre VEGF and sFlt-1 mutant mice at the end of the weaning period, we examined mice at 21 days to address a physiologically important time period by which VEGF regulation may have the greatest impact on postnatal gastrointestinal development. VEGF augmentation, as demonstrated by increased VEGF expression by RT-PCR and ELISA (Fig 1C and 1D), resulted in a deep red color of the intestine (Fig 1B), which has been previously reported in transgenic mice that overexpressed VEGF via the villin promoter [14]. The deeper red color is due to increased vascular leak and angiogenesis. VEGF mutant villi demonstrated a significant increase in single RBCs seen outside of the villus vasculature on H&E suggestive of increased vascular permeability or leak (Fig 4B and 4C), which has been reported in other models overexpressing VEGF [14, 36]. Three-dimensional volume reconstruction of FITC-dextran labeled vasculature demonstrated increased FITC-dextran volume within VEGF mutant villi (Fig 4F and 4G) supporting enhanced angiogenesis and vascular leak. Taken together with increased VE-cadherin expression by RT-PCR (Fig 5), there is a notable increase in vascular permeability and angiogenesis in VEGF mutants. Although previous studies of VEGF augmentation in the small intestine during embryonic development resulted in the development of epithelial cysts within the crypts and an increased frequency of intestinal adenomas, we did not appreciate similar results in our postnatal model [14]. However, postnatal VEGF overexpression in the colon demonstrated epithelial cysts throughout (S4 Fig). The discordance between the phenotype seen in small intestine versus colon in our model may result from changes in VEGFR expression levels during postnatal development. We confirmed that in mice, VEGFR2 expression by immunofluorescence is greater in colonic epithelium compared to the small intestine (S5 Fig). As a result, we speculate that VEGF augmentation may play a more significant role in the development of intestinal neoplasia and alteration of stem/progenitor cell populations during the postnatal period in the colon versus the small intestine. Our inducible VEGF model may be an important tool for further studies to investigate possible mechanisms by which VEGF promotes tumorgenesis in the adult colon.

Decreased VEGF-A bioavailability was achieved by overexpressing sFlt-1 in the brush border of intestinal epithelium using a doxycycline-inducible tet(O) expression of sFlt-1 and rtTA expression of Villin-Cre in enterocytes. The sFlt-1 mutants were significantly smaller in size, which has been reported in transgenic mice that reduce VEGF bioavailability in the gastrointestinal tract by overexpressing sFlt-1 in the mesenchyme [35]. Villin sFlt-1 mutant mice developed a shorter intestine and underdeveloped cecum (S1B–S1D Fig). Further histological evaluation of the cecum revealed a significant decrease in the number of goblet cells (S1E and S1F Fig); however, we did not appreciate a notable difference in goblet cells within the small intestine. VEGF induces Dll4 expression in endothelial tip cells [37]. Dll1 and Dll4 are expressed in distinct patterns along the crypt-villus axis of the gastrointestinal tract with significantly less Dll1 expression and greater Dll4 expression in colonic crypts compared to small intestine [38]. Loss of Notch signaling within intestinal epithelium via Dll1 and Dll4 blockade result in complete conversion of proliferating progenitors into goblet cells with a concurrent loss of intestinal stem and progenitor cells within the crypts of small intestine and colon [39]. It is therefore surprising that we would see a loss of goblet cells within the cecum of sFlt-1 mutant mice and no appreciable effect in goblet cell populations within the small intestine. These findings could implicate changes in notch signaling ligand and receptor expression within intestinal stem/progenitor cells that are reflective of the postnatal period compared to embryonic development.

RT-PCR expression of sFlt-1 was significantly decreased in sFlt-1 mutant duodenum after 21 days of doxycycline treatment (Fig 1G) compared to littermates with no significant change in VEGF-A transcript or protein levels (S1G and S1H Fig). These data suggest a compensatory negative feedback relationship wherein chronic sFlt-1 overexpression decreases VEGFR1 expression, as the RT-PCR primer used can amplify both sFlt-1 and full length Flt-1. VEGF-A levels within the native duodenum were significantly low, which is likely why we were unable to see a significant reduction in VEGF-A by ELISA. Therefore, to confirm that sFlt-1 overexpression within the intestinal epithelium does indeed occur in our transgenic model, enteroid cultures were created from sFlt-1 mutant mice. After administration of doxycycline, a significant increase in sFlt-1 expression was demonstrated without altering expression of KDR, another VEGF receptor (Fig 1H and S2A Fig). Taken together, the molecular and phenotypic evidence supports that villin-positive cell overexpression of sFlt-1 leads to reduced VEGF-A bioavailability in sFlt-1 mutants, similar to our previously described mesenchymal-expressed sFlt-1 murine model [35].

The murine postnatal period is important for the maturation of the intestine as upward migration of the crypt-villus axis promotes mature crypts formation in intervillus pockets, giving rise to intestinal stem cells [40, 41]. Histological analysis of VEGF mutants revealed increased villus height, but no change in crypt depth (Fig 2). In contrast, sFlt-1 mutants exhibited decreased villus height with longer crypts (Fig 3). In swine, increased vascular flow rate and resistance is crucial for normal development of villi during the postnatal period [42]. The discordance in angiogenesis seen between VEGF and sFlt-1 mutant mice and its inherent effects on vascular flow and resistance might account for the significant changes in villus and crypt architecture; however, there is likely a direct effect of VEGF and additional angiogenic/vasculogenic signaling molecules on epithelial and other cell types. Platelet-derived growth factor (PDGF) acts with some coordination with VEGF in angiogenesis and contributes to proper crypt morphology and its inhibition leads to fewer and misshapen crypts [43]. VEGF-A binds to PDGF receptors and induces signaling in mesenchymal cells [44]. Therefore, the increased villus height in VEGF mutants may be occurring through PDGF receptor signaling in the small intestine mesenchyme, aiding in the formation and elongation of villi. This is in contrast to sFlt-1 mutant mice, which demonstrated shorter villi with longer crypts compared to littermates. As the crypt-villus axis migrates upward during crypt formation, the villi are shorter, but the crypts elongate, suggesting that VEGF could play a role in crypt-villus axis development and migration by directly regulating epithelium gene expression or indirectly through the mesenchyme.

VEGF augmentation and reduction had antagonistic effects on the number and location of proliferating cells compared to littermate controls. In VEGF mutants, Ki-67-positive cells were greater in number and extended higher into the transit-amplifying zone compared to littermates (Fig 6) and was not influenced by any appreciable induction of apoptosis via caspase 3 (S3 Fig). Absorptive and secretory progenitor cells typically reside in this region, though no change in Hes1 or Atoh1 RNA expression was identified. Therefore, Ki-67-positive cells may be proliferating under the direction of secondary mediators of VEGF signaling, such as the Notch signaling ligands Dll1 and Dll4 that are required for maintenance and of intestinal stem/progenitor cells [39, 45]. Moreover, increased proliferation in VEGF mutants could be due to a reduction in epithelial cell migration along the crypt-villus axis [14]. In contrast, the sFlt-1 mutants displayed decreased proliferation in the transit-amplifying zone and an increase in the number of Paneth cells (Figs 6 and 7). Similar results of reduced body weight, shorter villus height and decreased proliferation of the transit-amplifying cells with an increase in Paneth cells have been identified in Krüppel-like factor 9 (Klf9)-deficient mice [46]. Several signaling molecules involved in vasculogenesis were downregulated in Klf9-deficient mice, indicating the complex interplay between the mesenchyme and its effects on migration and proliferation of the vasculature, epithelium and intestinal stem/progenitor cell populations [46]. These data also suggest a potential supportive role of angiogenic development and proper maintenance of intestinal stem/progenitor cell progenitor homeostasis.

RT-qPCR identified differences in intestinal stem cell gene expression in VEGF and sFlt-1 mutants. Several experimental and biological limitations are encountered when performing gene analysis by RT-qPCR, including a discordance between transcript expression and protein translation [47]. Therefore, downstream effectors with significant changes in expression still need to be identified, but the effect at the RNA transcription level suggests an important role of VEGF signaling in the maintenance and regulation of the intestinal stem cell niche. Lgr5 cells are rapidly cycling and sensitive to irradiation, while Bmi1 cells are slow-cycling and more quiescent. VEGF mutants had decreased Lgr5 expression, but no change in expression of Bmi1 (Fig 8). Lgr5 reduction in the VEGF mutants did not alter the differentiated cell types in the intestinal epithelium, possibly due to replacement by differentiating Bmi1 cells which can compensate for the loss of Lgr5-positive cells [48, 49]. In contrast, sFlt-1 mutants had no change in Lgr5 expression, but a 0.42-fold decrease in Bmi1 expression compared to littermates (Fig 8). The sFlt-1 mutants display a number of characteristics that can be partially explained by decreased Bmi1 expression. *Bmi1* knockout mice have shorter small intestines, similar to that in our sFlt-1 mutant mice [50]. In sFlt-1 mutants, decreased proliferation per hemicrypt is observed. Similarly, the expression of Wdr43, a marker of transit-amplifying cells, was 0.60-fold less in sFlt-1 mutants than littermates. This decrease is consistent with the lower percentage of Ki-67-positive cells identified in the transit-amplifying zone of the crypt. While Bmi1 expression was decreased in the sFlt-1 mutants, the expression of Lgr5 was unaffected. VEGF-induced expression of Dll4 in vascular endothelium leads to the activation of Notch signaling [39, 51]. With reduced bioavailability of VEGF-A, Notch signaling potentially decreases, resulting in decreased Bmi1 and Wdr43 expression with a subsequent reduction in proliferation within the crypts of the small intestine. VEGF/neuropilin-2 signaling as been shown to repress insulin-like growth factor-1 receptor expression through Bmi-1 [52]. Elevated insulin receptor B (IR-B) levels in intestinal epithelial stem cells decreased proliferation in the crypts and enhanced epithelial barrier function [53]. VEGF reduction could result in decreased expression of Bmi-1 and a subsequent increase in IR-B receptor signaling, leading to decreased intestinal stem cell proliferation and subsequent increase in Paneth cell differentiation within the crypts.

The sFlt-1 mutants were found to have a 1.5-fold increase in Sox9 expression (Fig 8). Sox9 expression inhibits proliferation *in vivo*[26], which may have contributed to the decrease in the number of proliferating cells per hemicrypt, resulting in decreased villus height. Highly expressed in Paneth cells, Sox9 controls an early step in Paneth cell differentiation through interactions with the Wnt signaling pathway and affects the overall phenotype of the small intestine crypts. Consistent with the increase in Sox9 expression, there was an increase in the percentage of lysozyme-positive cells per hemivillus in the sFlt-1 mutants (Fig 8). Insulin-like growth factor 1 has been shown to enhance crypt regeneration and increase the percentage of intestinal stem cells in S-phase without expanding the population [54]. Thus, IGF signaling primes intestinal stem cells to differentiate during conditions of cell loss or injury. Moreover, SOX9 has also been shown to be a transcriptional regulator of insulin-like growth factor-binding protein 4 (IGFBP-4), which is expressed highly in Paneth cells [55]. SOX9-induced activation of IGFBP-4 is directly involved in the antiproliferative effects seen on intestinal stem/progenitor cells and may explain the phenotype we observe in sFlt-1 mutants as loss of VEGF signaling may induce increased expression of IGF-IR through Bmi1, thereby promoting Paneth cell differentiation and intestinal crypt regeneration.

The sFlt-1 mutants also demonstrated a significantly increased expression of EphB2 (Fig 7). EphB2 expression is under control of the Wnt pathway and is usually highly expressed in intestinal stem cells, with decreased amounts in more differentiated cells [27]. Increased EphB2 is associated with increased proliferation and promotion of cell cycle re-entry. It is also found to be increased in wound healing in inflamed intestines [56]. The increased expression of Sox9 and EphB2 and the decrease in proliferating cells is consistent with the ability of these progenitor cells to self-renew and differentiate into diverse cell types.

In both VEGF and sFlt-1 mutant mice, there was no significant difference in expression of Atoh1 and Hes1, which mark progenitor cells in the transit-amplifying zone and regulate differentiation into secretory cell fates. Atoh1 is important in differentiation of intestinal stem cells into secretory cell fates, whereas Hes1 has been shown to promote proliferation and inhibit secretory cell development [57, 58]. We did not identify notable differences in the ability of VEGF or sFlt-1 mutants to differentiate into secretory cells lineages in the small intestine.

To isolate the effects of VEGF signaling on epithelial cells without confounding interactions from native intestinal vasculature, OU were obtained from VEGF mutants and grown in culture over a period of 10 days. OU are comprised of epithelium and mesenchyme and are devoid of a vascular supply. We have demonstrated expression of VEGFR2 (Flk) within the epithelium of OU *in vitro* (S5E Fig), suggesting that VEGF may have direct signaling effects on intestinal epithelium. Additionally, Caco-2 human intestinal epithelial cells are known to express VEGF receptors [30]. Although VEGF mutant OU exposed to doxycycline were larger in diameter at day 5 and demonstrated a significant increase in VEGF protein expression, differences in OU diameter were not maintained at day 10 *in vitro* (Fig 9A and 9B). To identify if the discordance in size at day 5 resulted from changes in stem/progenitor cell populations, we examined several stem cell markers by RT-PCR. Increased expression of Bmi1 and Atoh1 occurred, whereas EphB2 was downregulated in doxycycline-treated VEGF OU compared to controls at 5 days (Fig 9C). Significant changes in stem/progenitor cell marker expression were lost by 10 days *in vitro* (S2D Fig). Bmi1-positive slow-cycling ISCs have the capacity to self-renew, proliferate, and give rise to all the differentiated epithelial cell lineages of the small intestine [22, 48–50]. EphB2 expression within the intestinal crypt is highly expressed in ISCs and decreases as cells proliferate and contribute to the transit-amplifying zone [27, 40]. In VEGF OU, upregulation of Bmi1 and concurrent downregulation of EphB2 suggest that VEGF stimulates ISCs to proliferate and migrate within the transit-amplifying zone, supportive of increased Ki67-positive cells within the transit-amplifying zone of VEGF mutant mice at 21 days. Atoh1 upregulation suggests that VEGF may prime progenitor cells within the transit-amplifying zone to preferentially differentiate into cells of the secretory lineage [24]. Our *in vitro* data suggest that the increase in epithelial cell proliferation seen in the transit-amplifying zone of VEGF mutant duodenum could represent a direct effect by VEGF on epithelial stem/progenitor cells, independent of effects from the native vasculature. However, the notable discordance between stem/progenitor cell gene expression between VEGF mutant duodenum at 21 days and VEGF OU at 5 days may occur primarily due to acute versus chronic VEGF exposure or secondary effects from native intestinal vasculature *in vivo*. Further exploration using OU culture as a system to discern between primary effects of VEGF on the intestinal epithelium and contributory secondary effects mediated through the native vasculature at earlier timepoints is needed. Furthermore, VEGF and sFlt-1 mutant mice demonstrate distinct phenotypic differences in stem/progenitor cells and terminally differentiated cell types between the small intestine and colon at the end of the weaning period. Elucidation of the mechanisms that account for differences seen between small intestine and colon using our inducible *in vivo* and OU culture models will provide a useful strategy to identify regulators of intestinal stem cell/progenitor cell maintenance, homeostasis and differentiation in discrete areas of the gastrointestinal system.

Alterations in VEGF bioavailability had unique effects on postnatal small intestinal development in a murine model. Overexpression of VEGF led to increased angiogenesis and vascular permeability as well as increased proliferation higher into the transit-amplifying zone. *In vivo*, VEGF augmentation was found to decrease expression of Lgr5 without affecting expression of Bmi1. In OU culture, VEGF augmentation led to increased expression of Bmi1 and Atoh1 with a reciprocal downregulation of EphB2, suggesting primary effects of VEGF on intestinal epithelial stem/progenitor cell maintenance and homeostasis. *In vivo*, sequestration of VEGF caused inhibited vasculogenesis and restricted proliferation of intestinal epithelial cells within the crypts. Decreased availability of VEGF led to increased expression of Sox9 and EphB2, as well as a concomitant decrease in expression of Bmi1 and Wdr43. Future studies using concurrent *in vivo* and OU culture models to explore the role of VEGF signaling during early postnatal development may provide a useful platform to distinguish between the direct and indirect mechanisms by which VEGF bioavailability affects intestinal stem/progenitor cell populations and alters postnatal intestinal development.

## Supporting Information

S1 Fig Phenotype of sFlt-1 mutant mouse, RT-PCR and ELISA of VEGF in duodenum. (A) sFlt-1 mutant demonstrate decreased body size and appeared to have swollen anuses after 21 days of induction with doxycycline (B) The sFlt mutant gastrointestinal tract is shorter in length with a diminutive cecum (red box) compared to littermates (blue box). (C) H&E staining of littermate cecum. (D) H&E staining of mutant cecum demonstrating a decrease in secretory cells. (E) Immunofluorescence staining of goblet cells in littermate cecum; Mucin (Muc2, Red); Nuclei (DAPI, Blue). (E) sFlt cecum demonstrates less goblet cells as compared to littermates. (F) RT-PCR of VEGF-A in sFlt mutant duodenum demonstrated no significant change in VEGF expression (p = 0.6). N = 3 mice per group. Error Bars SEM. (G) RT-PCR of VEGF-A in sFlt mutant enteroid culture demonstrated no significant change in VEGF expression after doxycycline treatment (p = 0.88). N = 3 mice per group. Error Bars = SEM.
(TIFF)

S2 Fig VEGF mutant enteroid/OU culture and C57/B6 OU culture. **(A)** Doxycycline addition did not alter the expression of VEGFR2 (KDR) (p = 0.85) in VEGF OU. (B) VEGF mutant enteroid cultures are devoid of endothelial cells as compared to small intestine (*p< 0.001). (C) Doxycycline administration on wildtype C57/B6-derived OU demonstrates no significant change in size over 5 days *in vitro*. (D) Doxycycline-treated VEGF OU do not demonstrate significant differences in expression of stem/progenitor cell markers at 10 days. N = 25 OU per well, 6 wells; Error bars = SEM.
(TIFF)

S3 Fig Caspase 3 activation did not increase in VEGF or sFlt-1 mutant mice. Caspase 3 (Red) immunofluorescence staining of VEGF mutant duodenum (B) compared to littermates (A). Caspase 3 immunofluorescence staining of sFlt-1 mutant duodenum (D) compared to littermates (C). Nuclei were stained with DAPI (Blue). N = 4 mice per group.
(TIFF)

S4 Fig VEGF mutant mice demonstrate increased epithelial cysts within the colon. H&E sections of the distal colon in VEGF mutant mice had a predominance of epithelial cysts within the mucosa (black arrow). Cysts within the proximal colon were smaller in size and less frequent. Epithelial cysts were not identified in littermate controls. Alcian Blue staining did not demonstrate an appreciable change in goblet cell numbers.
(TIFF)

S5 Fig Flk staining in small intestine, colon and organoid units. (A) Immunofluorescence staining of E-cadherin (Green), Flk (Red) and DAPI (Blue) demonstrates colocalization of Flk with epithelial cells of the crypt and villus in the small intestine. (B) Flk staining is more prominent in the intravillus mesenchyme and vasculature (white outline) than in villus epithelial cells in small intestine, which more strongly stains the basal than apical surface. White arrows identify villus crypts. (C) Immunofluorescence staining of E-cadherin (Green), Flk (Red) and DAPI (Blue) demonstrates colocalization of Flk within epithelial cells of colon. (D) Flk/E-cadherin staining is prominent in the intercrypt epithelium (ICE, white outline). Colonic crypts (CC) have less Flk staining compared to epithelial cells within the ICE. Within the mesenchyme (M), we identify Flk-positive/E-cadherin-negative cells, which likely represent underlying vasculature. (E) OU culture demonstrates prominent colocalization of E-cadherin (Green) and Flk (Red) within the epithelium. (F) Positive control immunofluorescence staining of Flk (Red) in the hepatic artery (bottom left inset) and portal vein (white dotted outline and upper right inset) demonstrates Flk-positive endothelial staining. The hepatic artery more strongly expresses Flk compared to the portal vein, which has been previously described and may explain the differences in levels of Flk staining seen in epithelium of small intestinal villi and crypts as compared to the colon. Scale bars = 100 μm.
(TIFF)
