# Supplementary figures and images for: Vascular Endothelial Growth Factor (VEGF) Bioavailability Regulates Angiogenesis and Intestinal Stem and Progenitor Cell Proliferation during Postnatal Small Intestinal Development
Source: PLoS One. 2016 Mar 15;11(3):e0151396. doi: 10.1371/journal.pone.0151396 (PMC4792464; doi:10.1371/journal.pone.0151396)

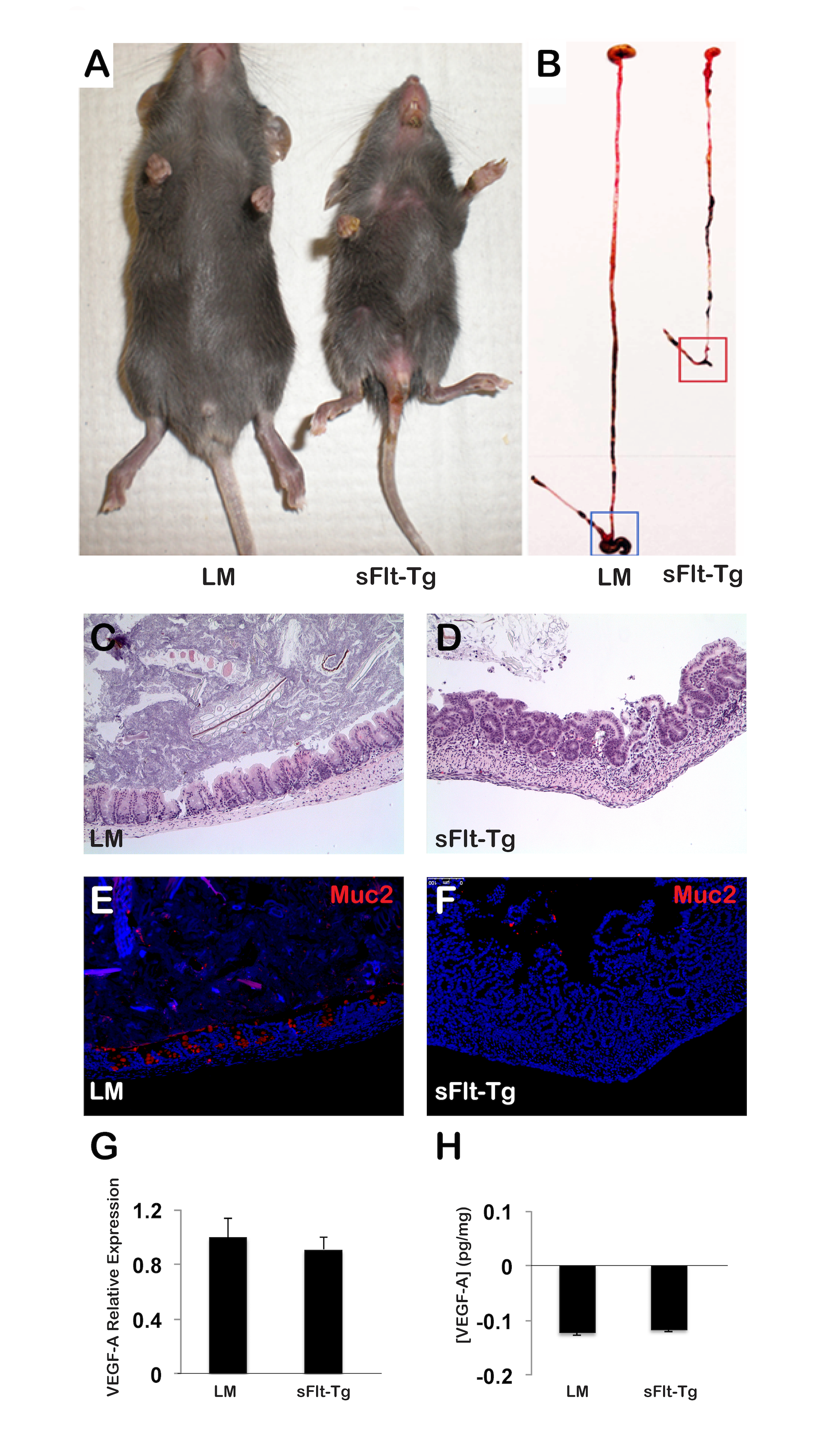

Supplement: S1 Fig — (A) sFlt-1 mutant demonstrate decreased body size and appeared to have swollen anuses after 21 days of induction with doxycycline (B) The sFlt mutant gastrointestinal tract is shorter in length with a diminutive cecum (red box) compared to littermates (blue box). (C) H&E staining of littermate cecum. (D) H&E staining of mutant cecum demonstrating a decrease in secretory cells. (E) Immunofluorescence staining of goblet cells in littermate cecum; Mucin (Muc2, Red); Nuclei (DAPI, Blue). (E) sFlt cecum demonstrates less goblet cells as compared to littermates. (F) RT-PCR of VEGF-A in sFlt mutant duodenum demonstrated no significant change in VEGF expression (p = 0.6). N = 3 mice per group. Error Bars SEM. (G) RT-PCR of VEGF-A in sFlt mutant enteroid culture demonstrated no significant change in VEGF expression after doxycycline treatment (p = 0.88). N = 3 mice per group. Error Bars = SEM. (TIFF) [file pone.0151396.s001.tiff]

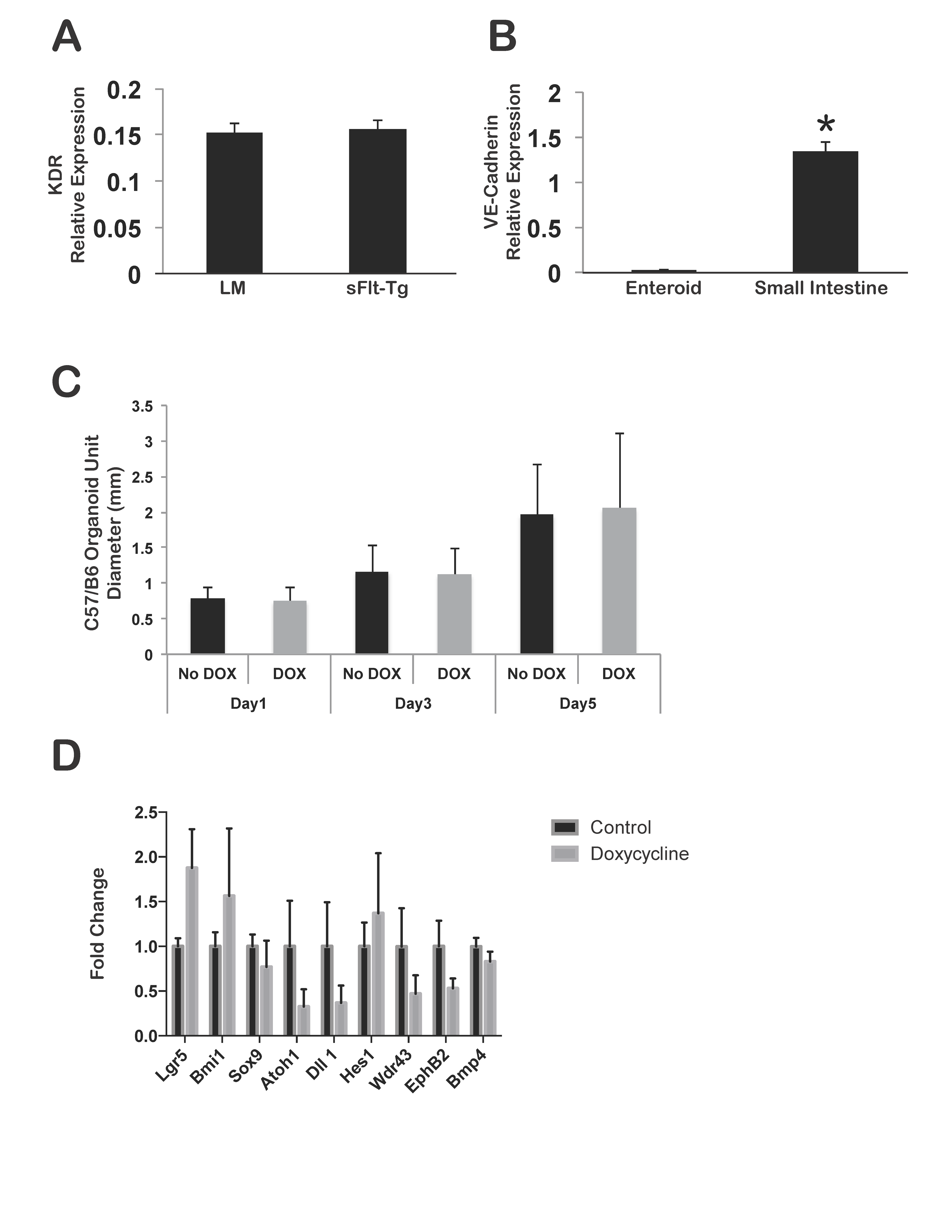

Supplement: S2 Fig — (A) Doxycycline addition did not alter the expression of VEGFR2 (KDR) (p = 0.85) in VEGF OU. (B) VEGF mutant enteroid cultures are devoid of endothelial cells as compared to small intestine (*p< 0.001). (C) Doxycycline administration on wildtype C57/B6-derived OU demonstrates no significant change in size over 5 days in vitro. (D) Doxycycline-treated VEGF OU do not demonstrate significant differences in expression of stem/progenitor cell markers at 10 days. N = 25 OU per well, 6 wells; Error bars = SEM. (TIFF) [file pone.0151396.s002.tiff]

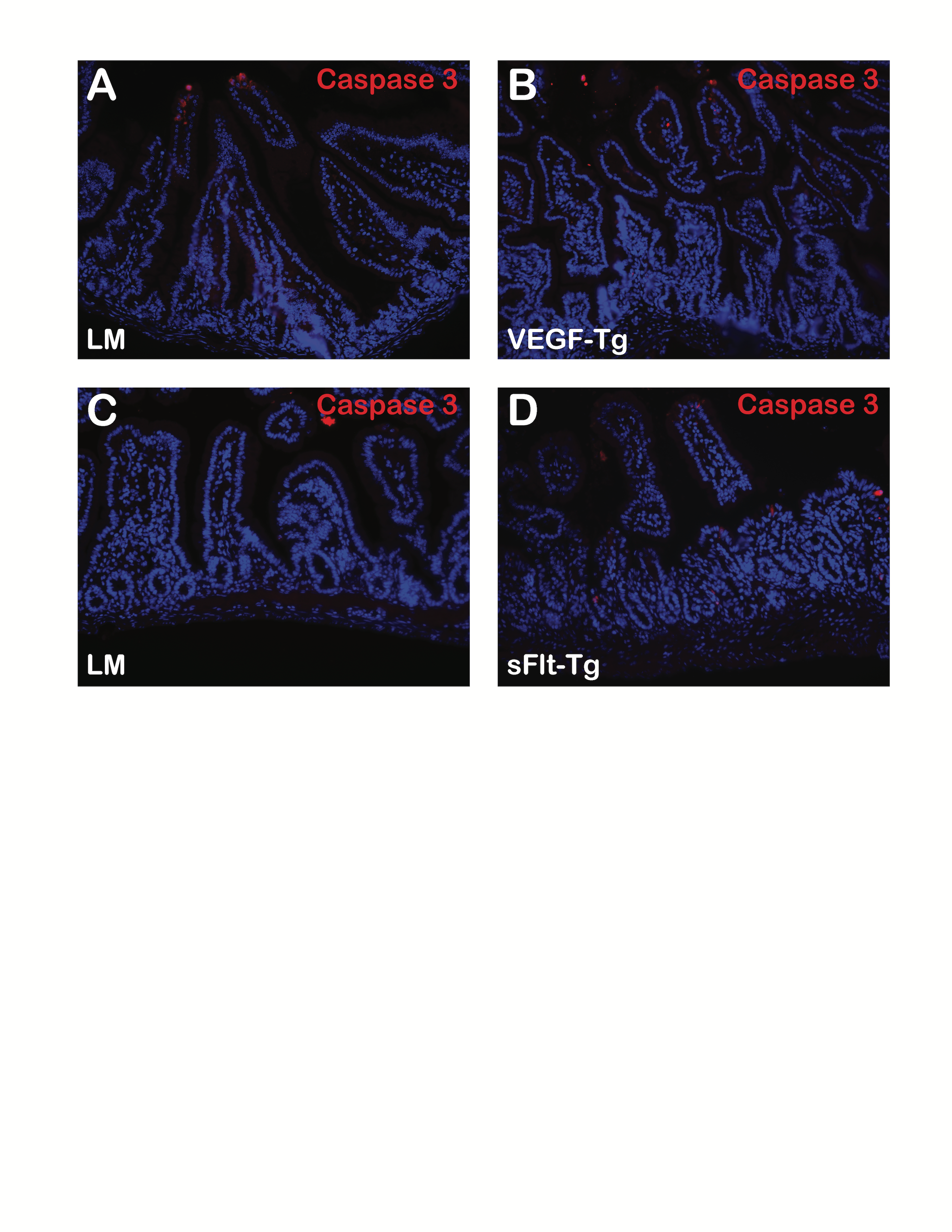

Supplement: S3 Fig — Caspase 3 (Red) immunofluorescence staining of VEGF mutant duodenum (B) compared to littermates (A). Caspase 3 immunofluorescence staining of sFlt-1 mutant duodenum (D) compared to littermates (C). Nuclei were stained with DAPI (Blue). N = 4 mice per group. (TIFF) [file pone.0151396.s003.tiff]

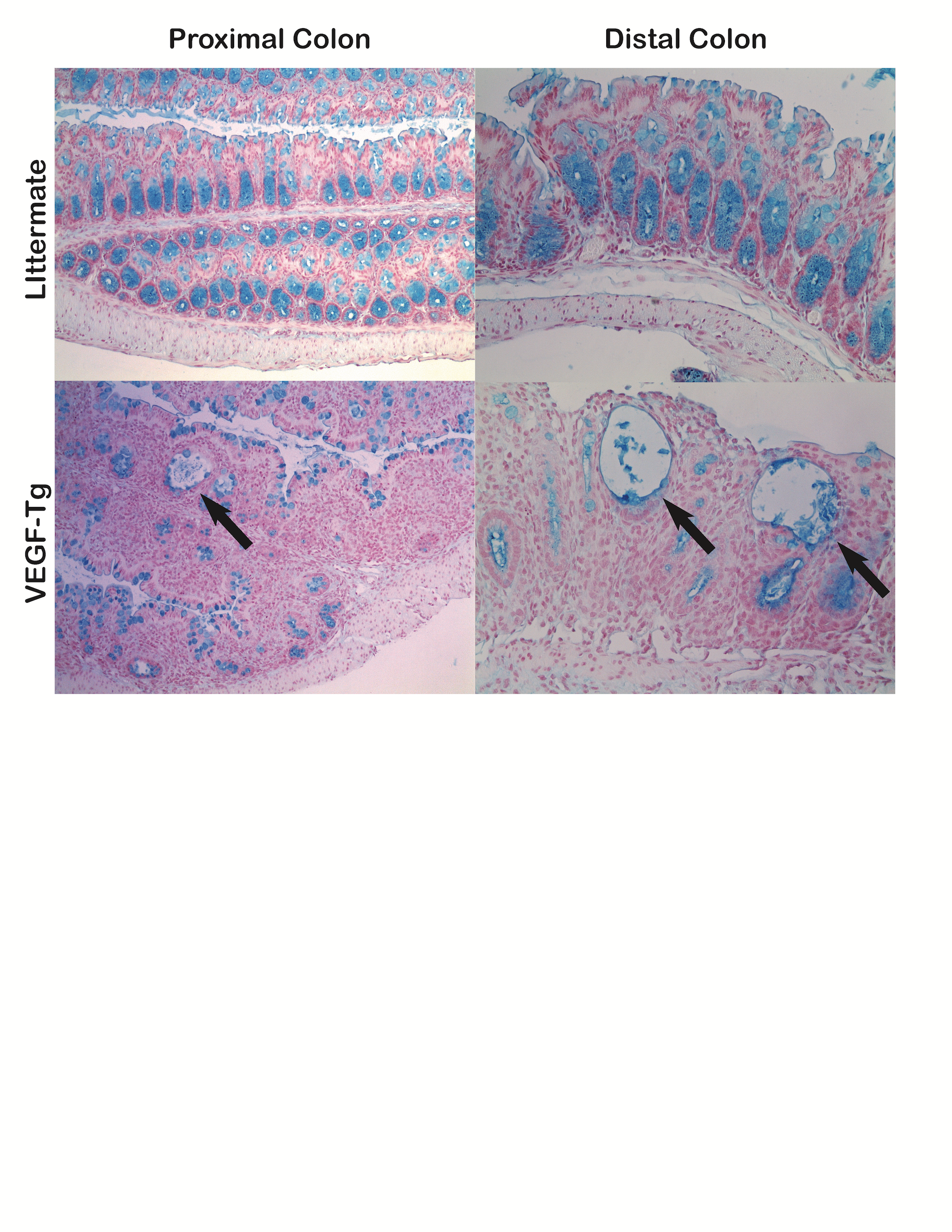

Supplement: S4 Fig — H&E sections of the distal colon in VEGF mutant mice had a predominance of epithelial cysts within the mucosa (black arrow). Cysts within the proximal colon were smaller in size and less frequent. Epithelial cysts were not identified in littermate controls. Alcian Blue staining did not demonstrate an appreciable change in goblet cell numbers. (TIFF) [file pone.0151396.s004.tiff]

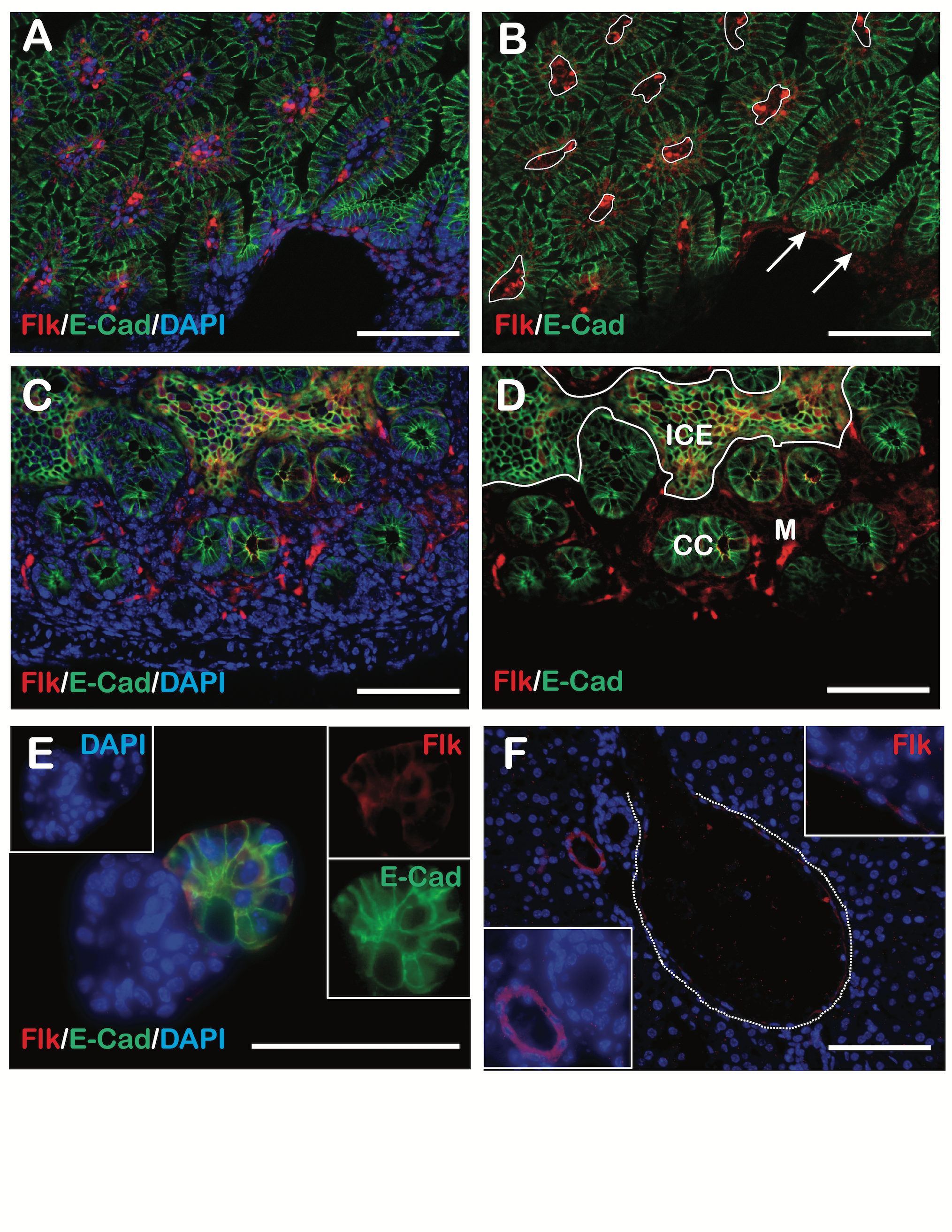

Supplement: S5 Fig — (A) Immunofluorescence staining of E-cadherin (Green), Flk (Red) and DAPI (Blue) demonstrates colocalization of Flk with epithelial cells of the crypt and villus in the small intestine. (B) Flk staining is more prominent in the intravillus mesenchyme and vasculature (white outline) than in villus epithelial cells in small intestine, which more strongly stains the basal than apical surface. White arrows identify villus crypts. (C) Immunofluorescence staining of E-cadherin (Green), Flk (Red) and DAPI (Blue) demonstrates colocalization of Flk within epithelial cells of colon. (D) Flk/E-cadherin staining is prominent in the intercrypt epithelium (ICE, white outline). Colonic crypts (CC) have less Flk staining compared to epithelial cells within the ICE. Within the mesenchyme (M), we identify Flk-positive/E-cadherin-negative cells, which likely represent underlying vasculature. (E) OU culture demonstrates prominent colocalization of E-cadherin (Green) and Flk (Red) within the epithelium. (F) Positive control immunofluorescence staining of Flk (Red) in the hepatic artery (bottom left inset) and portal vein (white dotted outline and upper right inset) demonstrates Flk-positive endothelial staining. The hepatic artery more strongly expresses Flk compared to the portal vein, which has been previously described and may explain the differences in levels of Flk staining seen in epithelium of small intestinal villi and crypts as compared to the colon. Scale bars = 100 μm. (TIFF) [file pone.0151396.s005.tiff]
